# A Novel Computational Model of the Rabbit Atrial Cardiomyocyte With Spatial Calcium Dynamics

**DOI:** 10.3389/fphys.2020.556156

**Published:** 2020-10-09

**Authors:** Márcia R. Vagos, Hermenegild Arevalo, Jordi Heijman, Ulrich Schotten, Joakim Sundnes

**Affiliations:** ^1^Simula Research Laboratory, Computational Physiology Department, Lysaker, Norway; ^2^Department of Informatics, University of Oslo, Oslo, Norway; ^3^Center for Cardiological Innovation, Rikshospitalet, Oslo, Norway; ^4^Faculty of Health, Medicine and Life Sciences, CARIM School for Cardiovascular Diseases, Maastricht University, Maastricht, Netherlands

**Keywords:** rabbit atrial cardiomyocyte, computational model, spatial calcium dynamics, calcium waves, population of models, correlation analysis

## Abstract

Models of cardiac electrophysiology are widely used to supplement experimental results and to provide insight into mechanisms of cardiac function and pathology. The rabbit has been a particularly important animal model for studying mechanisms of atrial pathophysiology and atrial fibrillation, which has motivated the development of models for the rabbit atrial cardiomyocyte electrophysiology. Previously developed models include detailed representations of membrane currents and intracellular ionic concentrations, but these so-called “common-pool” models lack a spatially distributed description of the calcium handling system, which reflects the detailed ultrastructure likely found in cells *in vivo*. Because of the less well-developed T-tubular system in atrial compared to ventricular cardiomyocytes, spatial gradients in intracellular calcium concentrations may play a more significant role in atrial cardiomyocyte pathophysiology, rendering common-pool models less suitable for investigating underlying electrophysiological mechanisms. In this study, we developed a novel computational model of the rabbit atrial cardiomyocyte incorporating detailed compartmentalization of intracellular calcium dynamics, in addition to a description of membrane currents and intracellular processes. The spatial representation of calcium was based on dividing the intracellular space into eighteen different compartments in the transversal direction, each with separate systems for internal calcium storage and release, and tracking ionic fluxes between compartments in addition to the dynamics driven by membrane currents and calcium release. The model was parameterized employing a population-of-models approach using experimental data from different sources. The parameterization of this novel model resulted in a reduced population of models with inherent variability in calcium dynamics and electrophysiological properties, all of which fall within the range of observed experimental values. As such, the population of models may represent natural variability in cardiomyocyte electrophysiology or inherent uncertainty in the underlying experimental data. The ionic model population was also able to reproduce the U-shaped waveform observed in line-scans of triggered calcium waves in atrial cardiomyocytes, characteristic of the absence of T-tubules, resulting in a centripetal calcium wave due to subcellular calcium diffusion. This novel spatial model of the rabbit atrial cardiomyocyte can be used to integrate experimental findings, offering the potential to enhance our understanding of the pathophysiological role of calcium-handling abnormalities under diseased conditions, such as atrial fibrillation.

## 1. Introduction

Mathematical models of cardiac electrophysiology (EP) have advanced significantly over the past decades, and are valuable tools for gaining physiological insight from the expanding pool of experimental data (Heijman et al., 2016; Vagos et al., 2018). While animal models remain the primary source of experimental data on ion channels and electrical activity in the heart, computational models of different animal species constitute an important tool for knowledge extraction and translation between species. The rabbit has been a particularly useful animal model to study different aspects of cardiac electrophysiology and arrhythmia, given the similarities of their electrophysiological properties to the human (Ravelli and Allessie, 1997; Eijsbouts et al., 2003; Rouge et al., 2006; Greiser et al., 2014; Li et al., 2014; Wang et al., 2017; Frommeyer et al., 2019). The wide application of the experimental rabbit model has motivated the development of rabbit-specific mathematical models of cardiomyocyte (CM) electrophysiology (see e.g., Hilgemann and Noble, 1987; Demir et al., 1994, 1999; Lindblad et al., 1996; Kurata et al., 2002; Shannon et al., 2004; Mahajan et al., 2008; Aslanidi et al., 2009; Maltsev and Lakatta, 2009), which incorporate rabbit-specific formulations of ionic currents.

One important characteristic of rabbit atrial CMs is the lack of a well-developed T-tubule (TT) system (Tidball et al., 1991; Blatter, 2017), which leads to a characteristic U-shaped wave front in line scans of intracellular calcium, indicating asynchronous Ca^2+^ release (Smyrnias et al., 2010; Greiser et al., 2014). This shape results from the “fire-diffuse-fire” response (Coombes and Timofeeva, 2003), in which a Ca^2+^ wave is initiated by L-type Ca^2+^ channels (LTCC) at the cell periphery and subsequently propagates toward the center of the cell in a saltatory manner through diffusion and Ca^2+^-induced Ca^2+^ release (Bootman et al., 2002, 2011). Similar U-shaped Ca^2+^ propagation patterns have been observed in atrial CMs of other small animal species, such as cat (Hüser et al., 1996; Blatter et al., 2003), and rat (Mackenzie et al., 2004; Bootman et al., 2011). However, it has also been observed in rat atrial CMs that Ca^2+^ signals originating at the cell periphery typically did not fully propagate to the center (Bootman et al., 2011). This effective truncation of the Ca^2+^ wave was due to the lack of TTs, increased Ca^2+^ buffering capacity, and the so-called “diffusion barrier” of the mitochondria and Serca2a in these cells. This lack of regeneration of the Ca^2+^ signal results in a progressive damping of the centripetal Ca^2+^ wave with a peak amplitude and rate of Ca^2+^ rise significantly lower at central regions as compared to the periphery (Mackenzie et al., 2004; Trafford et al., 2013). These observations have also been replicated in a model of Ca^2+^ propagation in a CM without TTs, demonstrating that Ca^2+^ propagation or lack thereof results from a complex interplay between different effectors of the Ca^2+^ handling system (Thul et al., 2012). The reduced systolic Ca^2+^ levels and delayed Ca^2+^ signals in central regions associated with the lack of TTs in some atrial CMs, as well as remodeling-induced detubulation, can thus have a profound effect on electrophysiological changes driven by ionic current remodeling or β-adrenergic stimulation (Trafford et al., 2013).

Despite the important role that spatial intracellular Ca^2+^ dynamics can play in the generation and maintenance of aberrant electrical activity in atrial cells and tissues, previously developed mathematical models of the rabbit atrial CM do not incorporate spatial description of Ca^2+^ movement within the cell. Therefore, although useful in reproducing whole-cell characteristics of rabbit EP, these models are not able to assess the sub-cellular mechanisms of altered Ca^2+^ propagation and their role in arrhythmic activity. In contrast, models with spatial Ca^2+^ description would permit assessment of the effect of sub-cellular structures on intracellular Ca^2+^ dynamics and Ca^2+^ wave propagation, but such models have not been developed for rabbit atrial physiology.

In this paper, we describe the development of a novel model of the rabbit atrial CM with spatial description of ionic species and the Ca^2+^ handling system. The structure of the model allows simulation of the spatial distribution of Ca^2+^, as well as the propagation of intracellular Ca^2+^ over time. We parameterized the maximum conductances in the model using a “population of models” approach to reproduce the normal electrophysiological properties of the rabbit, as supported by experimental data reported in the literature. The result is a population of models that all closely approximate the experimental data, but with differences in the models that appropriately reflect individual variability or inherent uncertainty in the data. We shall henceforth refer to this selected population as “control population.” We are aware that the dynamics of Ca^2+^ wave propagation are most directly affected by parameters that modulate the SR content, and Ca^2+^ release and uptake kinetics. However, the aim of the present study was not to focus on the role of these parameters in modulating Ca^2+^ wave propagation, but instead to parameterize the maximum conductances of ionic currents and asses their effect on intracellular Ca^2+^ dynamics Thus, the motivation to create a spatial model was to study the model's behavior in terms of this radial Ca^2+^ flux as we changed the maximum conductances of ion currents, and our primary hypothesis was that variations in ion-channel expression and function (in particular of Ca^2+^ currents) may contribute to alterations in radial Ca^2+^ flux. We then used the control population to (1) assess if changes in current conductances have an effect on Ca^2+^ propagation dynamics; (2) to quantify possible correlations between Ca^2+^ properties at the membrane and at the center of cell; and (3) to query the underlying mechanisms of the differences observed in the Ca^2+^ propagation patterns across the control population.

## 2. Methods

### 2.1. Model Development

The rabbit atrial CM model was developed based on the previously published human atrial CM model by Voigt & Heijman (Voigt et al., 2014), which is a spatial model with the calcium handling system divided into discrete domains. We modified this model to incorporate ion current formulations from the non-spatial rabbit atrial CM model by Lindblad et al. (1996) and Aslanidi et al. (2009) to reflect the rabbit electrophysiology.

**Figure 1:**
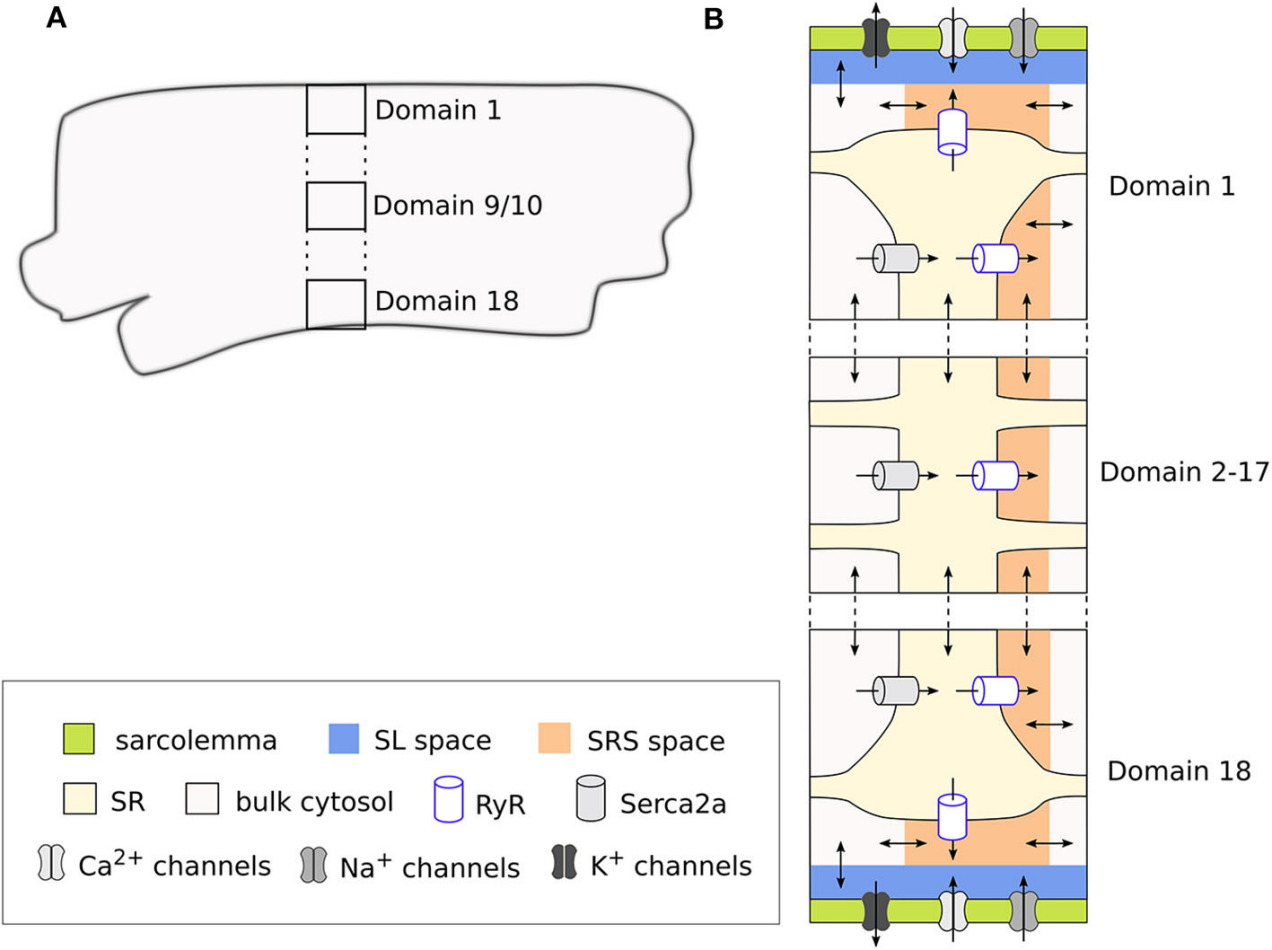
Schematic representation of a segment of the rabbit CM model showing the discrete cell domains and different intracellular compartments. A single segment corresponds to the cross section of the CM **(A)**, and is composed of 18 domains. Here only 3 domains are represented for simplicity, with the vertical dots representing the omitted domains. The membrane domains (1 and 18) contain the sarcolemmal currents and Ca^2+^ handling system, while the inner domains (2–17) contain only the Ca^2+^ handling system **(B)**. Each membrane domain contains five different compartments: cytosolic space, sarcoplasmic reticulum (SR), sub-SR (SRS) space, and subsarcolemmal (SL) spaces, which together constitute the cleft space. Ca^2+^ diffuses between the different compartments in the model, and between adjacent domains and segments. Because we use a deterministic RyR formulation, the model is symmetrical around the two central domains.

The model is structured into discrete segments in the longitudinal direction of the CM, and each segment is further divided into discrete domains in the radial (or transversal) direction. This architecture allows simulating the radial flux of Ca^2+^ from the membrane toward the center of the CM (i.e., centripetal diffusion) through an implementation of Ca^2+^ diffusion terms. Thus, a total of 18 domains represent a one-dimensional cross-section of the cell, where the two outermost domains (1 and 18) correspond to the region close to the cell membrane. These domains therefore include the sarcolemmal currents, Ca^2+^ release units (CRU), and Ca^2+^ buffers. In contrast, the inner domains (2–17) are not in direct contact with the cell membrane and contain only the CRUs and Ca^2+^ buffers. The inner domains contain a cytosolic space, the sarcoplasmic reticulum (SR), and a sub-SR space (SRS), while the membrane domains also include a subsarcolemmal (SL) space. In domains 1 and 18 the SRS represents a junctional space in which LTCC and the ryanodine receptors (RyR) interact, which is indicated with “junc.” This corresponds to the dyadic space in other CM models. We used a cell volume of 16 pL based on an estimated cell length of 130 μm (Lindblad et al., 1996) and a radius of 6.3 μm (Greiser et al., 2014). This volume is divided equally across the 18 domains, that the cytosolic, SR and SRS compartments make up 65, 3.5, and 0.1% of each domain, respectively, and that in addition the SL space takes up 2% of the cell volume. The domains and compartments, as well as the ionic fluxes between them, are schematically illustrated in Figure 1.

Because in this model Ca^2+^ enters the cell only through the membrane domains, CaTs in the inner domains are dependent on diffusion from the periphery. Thus, the model allows to reproduce radial gradients and possible heterogeneities in CaTs arising from local control mechanisms. The model also includes implementations of both deterministic and a stochastic RyR models. However, for the sake of simplicity, we have employed the deterministic formulation of the RyR, and thus in our simulations only one segment was simulated, since segments are identical when using the deterministic model.

The membrane model includes Ca^2+^ currents I_CaL_ and I_CaT_; the fast Na^+^ current I_Na_; repolarizing K^+^ currents I_to1_, I_Kr_, I_Ks_, and I_K1_, as well as three background currents I_Cab_, I_Nab_, and I_Clb_. Additionally, the model includes the ionic currents of the Na^+^-Ca^2+^ exchanger (I_NCX_); the Na^+^-K^+^ pump (I_NaK_); and the plasmalemmal Ca^2+^ pump (I_CaP_). The total membrane current in the model is the sum of all these currents:

$$\begin{array}{l} I_{\text{C}{\text{a}}_{\text{tot}}}=\left(I_{\text{Ca}{\text{L}}_{\text{junc}}}+I_{\text{Ca}{\text{L}}_{\text{SL}}}\right)+\left(I_{\text{Ca}{\text{T}}_{\text{junc}}}+I_{\text{Ca}{\text{T}}_{\text{SL}}}\right)+\left(I_{\text{Ca}{\text{P}}_{\text{junc}}}+I_{\text{Ca}{\text{P}}_{\text{SL}}}\right)+\\ +\left(I_{\text{Ca}{\text{b}}_{\text{junc}}}+I_{\text{Ca}{\text{b}}_{\text{SL}}}\right)-2\left(I_{\text{NC}{\text{X}}_{\text{junc}}}+I_{\text{NC}{\text{X}}_{\text{SL}}}\right). \end{array}$$

$$\begin{array}{l} I_{\text{N}{\text{a}}_{\text{tot}}}=\left(I_{\text{N}{\text{a}}_{\text{junc}}}+I_{\text{N}{\text{a}}_{\text{SL}}}\right)+3\left(I_{\text{NC}{\text{X}}_{\text{junc}}}+I_{\text{NC}{\text{X}}_{\text{SL}}}\right)-3\times \left(I_{\text{Na}{\text{K}}_{\text{junc}}}+I_{\text{Na}{\text{K}}_{\text{SL}}}\right)+\\ +\left(I_{\text{Na}{\text{b}}_{\text{junc}}}+I_{\text{Na}{\text{b}}_{\text{SL}}}\right). \end{array}$$

$$\begin{array}{l} I_{{\text{K}}_{\text{tot}}}=I_{\text{to}}+I_{\text{Kr}}+I_{\text{Ks}}+I_{\text{K}1}-2\times \left(I_{\text{Na}{\text{K}}_{\text{junc}}}+I_{\text{Na}{\text{K}}_{\text{SL}}}\right)+I_{\text{stim}}. \end{array}$$

$$\begin{array}{l} I_{\text{tot}}=I_{\text{C}{\text{a}}_{\text{tot}}}+I_{\text{N}{\text{a}}_{\text{tot}}}+I_{{\text{K}}_{\text{tot}}}+I_{\text{Clb}} \end{array}$$

$$\begin{array}{l} \frac{dV}{dt}=-\frac{1}{C_{\text{m}}}I_{\text{tot}} \end{array}$$

**Figure 2:**
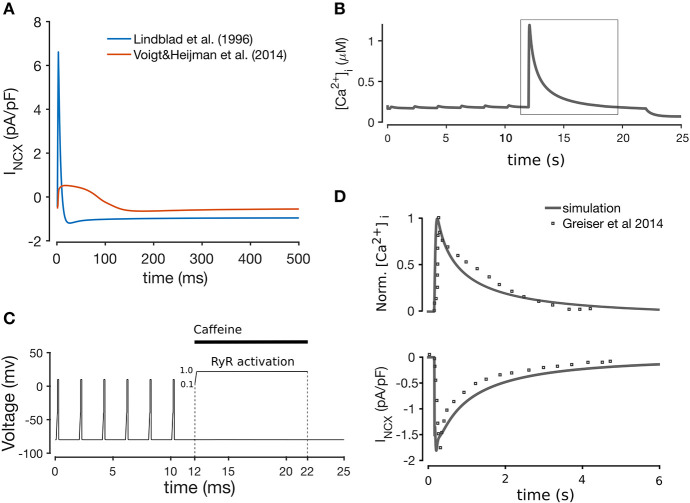
**(A)** Comparison between the Lindblad et al. (1996) and Voigt et al. (2014) models of the Na^+^-K^+^ exchanger, showing that the latter is closer to the I_NCX_ shape observed experimentally (Bers, 2002). Simulation of caffeine-induced Ca^2+^ release **(B)** using the voltage clamp protocol shown in **(C)**, whereby application of caffeine is simulated through the instant opening of the RyRs for 10 s with subsequent closing. **(D)** Comparison of I_NCX_ and normalized CaT with experimental data from Greiser et al. (2014), after pacing at 0.5 Hz for 12 s, and a 10 s quiescent period.

Most of the formulations were adopted from Aslanidi et al. (2009), which in turn are modifications of the formulations in Lindblad et al. (1996). We included the background chloride current (I_Clb_) from the Lindblad et al. model, since a chloride current has been reported in rabbit atrial CMs (Kanaporis and Blatter, 2016). The formulation of I_NCX_ was taken from Voigt et al. (2014) (equations provided in the Supplementary Material), originally described in Weber et al. (2001), since we found this current to replicate more realistically the forward and reverse modes of the Na^+^-Ca^2+^ exchanger observed in the rabbit Bers (2002), as shown in Figure 2A. We tested this model component specifically by simulating a caffeine-induced Ca^2+^ release protocol to assess Ca^2+^ extrusion rate through the NCX, shown in Figure 2B. The caffeine-induced Ca^2+^ release protocol used was as follows: the model was paced at 0.5 Hz (after being paced to steady state) for 12 s (6 beats), and then caffeine application was simulated by increasing RyR2 activation to 1.0 over the course of 10 ms, and keeping the channels fully open for another 10 s. The effect of the caffeine was then removed by unclamping the RyR activation for 3 s. This protocol is illustrated in Figure 2C. As illustrated in Figure 2D, the normalized CaT and decay time of I_NCX_ closely matched experimental data in Greiser et al. (2014).

The model's spatial description of Ca^2+^ and Na^+^ allows for these ionic species to vary between domains. Specifically, Ca^2+^ concentrations in the bulk cytosolic, SL, SR, and SRS spaces are updated independently in each compartment and in each model domain. Similarly, Na^+^ concentrations are updated separately in the SL, junc (SRS), and bulk cytosolic spaces, but the cytosolic Na^+^ trace is common to all domains, so that only this is updated in the inner domains. The concentrations and fluxes of Ca^2+^ and Na^+^ across the different model compartments in a membrane domain are schematically shown in Supplementary Figure 1. The mass balance equations for Ca^2+^ can be found in the supplementary material of Voigt et al. (2014), and the equations for Na^+^ are provided in the Supplementary Material.

The rabbit atrial CM model was implemented in C++, and the state equations were solved using a forward Euler scheme. All simulations were performed on the Abel computer cluster from the University of Oslo, running the Linux Operating system (64 bit CentOS 6). The source code of the model is available at https://github.com/marciavagos/Rabbit_model.git.

### 2.2. Parameterization of Ionic Currents

We initially implemented the model using the original published parameters from Aslanidi et al., which resulted in an action potential (AP) that was morphologically similar to experimentally measured APs, but did not match in terms of quantifiable metrics. The action potential duration at 90% repolarization (APD_90_) was 143 ms, which is longer than the reported 120 ms, and the resting membrane potential (RMP) was around −74 mV as compared to the −80 mV reported in literature. Additionally, the CaT amplitude (CaT-A) in the baseline model was only about 0.09 μM, compared to the ~1.0 μM amplitude in simulated CaTs by Lindblad et al. (1996), and Aslanidi et al. (2009). The model also showed a suppression of the Ca^2+^ signal at the center of cell. Ca^2+^ measurements of rabbit atrial CMs in Blatter (2017) show a partial suppression of the central CaT compared to the peripheral CaT. In contrast, measurements from Greiser et al. (2014) show a fully regenerative CaT in control rabbit atrial CMs. These differing findings seem to suggest a natural variability in the intracellular regeneration of the Ca^2+^ signal in rabbit atrial CMs. However, as far we know there is no evidence that complete absence of Ca^2+^ release in central regions is present in rabbit atrial CMs in the absence of disease-related remodeling, thus we considered this to represent an unphysiological behavior.

In order to adjust the model parameters to match reported experimental CaT and AP data (Qi et al., 1994; Muraki et al., 1995; Wang et al., 1995; Lindblad et al., 1996; Aslanidi et al., 2009; Greiser et al., 2014; Kanaporis and Blatter, 2016; Hou et al., 2018), we employed a population-of-models approach to scale the maximum conductances of the 13 ionic currents in the model. This approach is useful to perform parameter fitting, while at the same time allowing uncertainty and natural variability to be incorporated into the models (Amrita et al., 2012; Sánchez et al., 2014). We note that the populations-of-models approach is used in this work in a somewhat different context than done in previous works. While this approach has more commonly been used to incorporate variability into a “calibrated” model to either test hypotheses, or perform parameter sensitivity analysis (as done in e.g., Sarkar and Sobie, 2010; Amrita et al., 2012; Sánchez et al., 2014; Chang et al., 2015; Johnstone et al., 2016; Morotti and Grandi, 2017), here we have applied the method to parameterize the model in regard to uncertain parameters, in this case the maximum conductances. A population is constructed by randomly sampling the model parameters from specified probability distributions, thereby generating a population of several “baseline” models. For the present population, the 13 maximum conductances were varied over a range between 25 and 400% of their published values, and resampled from uniform distributions using Latin Hypercube sampling. Such a large degree of variation was chosen to incorporate as much variability as possible, allowing for the population to capture the natural variability and uncertainty in the data. The initial population consisted of 3,000 models, which were all paced at 2 Hz pacing for 2 min to ensure approximation to steady state, and then the last five beats were recorded for analysis. The varied maximum conductances and their respective nominal (published) values are listed in Table 1, and the initial ionic concentrations are listed in Supplementary Table 2.

**Table 1:** List of the maximum conductances of the ionic currents in the model and their nominal values.

**Parameter**	**Nominal value**	**Description**
G_**CaL**_	0.144 nS/pF	Maximum conductance of the L-type Ca^2+^ channel
G_CaT_	0.120 nS/pF	Maximum conductance of the T-type Ca^2+^ channel
${\text{I}}_{\text{NCX}}^{max}$	4.41 pA/pF	Maximum flux of the Ca^2+^-Na^+^ exchanger
${\text{I}}_{\text{NaK}}^{max}$	1.288 × 10−3 nA/pF	Maximum flux of the Na^+^-K^+^ pump
${\text{I}}_{\text{CaP}}^{max}$	0.190 nS/pF	Maximum flux of the plasmalemmal Ca^2+^ ATPase
G_Na_	0.028 × 10−3 µ*L*/(*spF*)	Maximum conductance of the fast Na^+^ channel
G_to_	0.200 nS/pF	Maximum conductance of the transient outward K^+^ channel
G_Kr_	0.070 nS/pF	Maximum conductance of the rapidly activating delayed rectifier K^+^ channel
G_Ks_	0.050 nS/pF	Maximum conductance of the slowly activating delayed rectifier K^+^ channel
G_K1_	0.203 nS/pF	Maximum conductance of the inward rectifier K^+^ channel
G_Cab_	0.4 × 10−3 nS/pF	Maximum conductance of the background Ca^2+^ channels
G_Nab_	0.4 × 10−3 nS/pF	Maximum conductance of the background Na^+^ channels
G_Clb_	2.4 × 10−3 nS/pF	Maximum conductance of the background K^+^ channels

Models in the population were then selected against experimental data from measurements of rabbit APs and CaTs to select models whose APs and CaTs represent typical rabbit-like morphology (Qi et al., 1994; Muraki et al., 1995; Wang et al., 1995; Lindblad et al., 1996; Aslanidi et al., 2009; Greiser et al., 2014; Kanaporis and Blatter, 2016; Hou et al., 2018).

Finally, uncertainties of output measures of APs and CaTs were defined through the mean and standard deviation (std).

### 2.3. Model Selection

Since we allowed a large degree of variation in the model parameters, the initial population included a number of non-physiological models. The second step of the population-of-models approach was to select a subset of models that matched previous experimental recordings, to create a control population. Experimentally measured APs of rabbit atrial CMs reported in literature are rather inconsistent. For instance, Muraki et al. (1995), and Wang et al. (1995) recorded APs with APD_90_ values of 93 and 103 ms, respectively, while Yamashita et al. (1995) reported 70 ms. Lindblad et al. (1996) reported a similar APD_90_ of 80 ms at 2 Hz, while more recent studies have reported higher values. Greiser et al. (2014) measured APD_90_ in rabbit CMs paced at 2 Hz between 100 and 140 ms, in agreement with the 130 ms Hou et al. (2018) at 1 Hz. APD_50_ measured in Wang et al. (1995) and Hou et al. (2018) was 44 (at 2 Hz) and 55 ms (at 1 Hz), respectively, while in Yamashita et al. (1995) this was about 18 ms in the crista terminalis, and 38 ms in pectinate muscle CMs. Additionally, APD_40_ in Qi et al. (1994) was 30 ms in left atrium, and 51 ms in right atrium at 1 Hz. APD_20_ in Hou et al. (2018) was also between 13 to 17 ms. Given this wide range of reported APD_50_ and APD_40_ values, we required APD_40_ to be between of 20 and 60 ms.

AP amplitude (APA) has been reported at around 100 mV (Qi et al., 1994; Muraki et al., 1995; Aslanidi et al., 2009; Hou et al., 2018), and 120 mV (Lindblad et al., 1996; Kanaporis and Blatter, 2016). Reported values of RMP are more consistent across sources, with most reporting around -80 mV (Qi et al., 1994; Muraki et al., 1995; Yamashita et al., 1995; Aslanidi et al., 2009; Greiser et al., 2014; Hou et al., 2018), although Lindblad et al. reported an RMP of -71 mV (Lindblad et al., 1996). Given this heterogeneity of measured electrophysiological properties of CMs, there is no single generic model of an atrial cell, which motivated our choice of a population-of-models approach to parameterize the model.

To our knowledge, absolute values of intracellular Ca^2+^ levels have only been reported by Kettlewell et al. (2019), who measured an average diastolic and systolic [Ca^2+^]_i_, and CaT amplitude of about 0.07, 0.57, and 0.51 μM, respectively. Additionally, Lindblad et al. (1996) assumed a resting Ca^2+^ of 50 nM, and peak Ca^2+^ levels in their model simulations were within ~0.1 and ~1.0 μM. CaTs measured from fluorescence imaging of rat (Mackenzie et al., 2001, 2004) and human (Voigt et al., 2012, 2014) atrial CMs also show amplitude values in this range. Therefore, we excluded models whose whole-cell CaT amplitude was outside this range. The rise time of the CaT was also constrained to be no larger than 100 ms at 2 Hz pacing (Greiser et al., 2014). Additionally, [Na^+^]_i_ was constrained to be between 6.5 and 12.5 mM, which corresponds to reported values in rabbit atrial cells (Hilgemann and Noble, 1987; Greiser et al., 2014). Finally, models showing early after-depolarizations (EAD) and alternans were also manually excluded.

These experimental metrics are compiled in Table 2 for convenience, and the output properties and corresponding value ranges considered in the selection of the models are listed in Table 3. These properties were determined as the average of the values calculated for the five recorded beats.

**Table 2:** Experimental values of electrophysiological parameters obtained from literature.

**Source**	**APD_**90**_ (**ms**)**	**APD_**50**_ (**ms**)**	**APD_**40**_ (**ms**)**	**APD_**20**_ (**ms**)**	**APA (**mV**)**	**RMP (**mV**)**	**CaT amp. (**µM**)**	**[Na^**+**^]_**i**_ (**mM**)**
Muraki et al. (1995)	93	–	–	–	~100	–	–	–
Wang et al. (1995)	103	44 (2 Hz)	–	–	–	–	–	–
Yamashita et al. (1995)	70	18 (CT) 38 (PM)	–	–	–	–	–	–
Lindblad et al. (1996)	80 (2 Hz) 55 (1 Hz)	–	–	–	–	−71	–	–
Qi et al. (1994)	–	–	30 (LA), 51 (RA) (1 Hz)	–	~100	–80	–	–
Aslanidi et al. (2009)	–	–	–	–	~100	−80	–	–
Voigt et al. (2012) (human)	–	–	–	–	–	–	[0.1–1]	–
Greiser et al. (2014)	[100–140] (2 Hz)				–	−80	–	[6.5–12.5]
Kanaporis and Blatter (2016)	–	–	–	–	~120	–	–	–
Hou et al. (2018)	~130	55 (1 Hz)	–	13–17	~100	−80	–	–
Kettlewell et al. (2019)	–	–	–	–	–	–	[0.1–1]	–
Mackenzie et al. (2004) (rat)	–	–	–	–	–	–	[0.1–1]	–
Hilgemann and Noble (1987)	–	–	–	–	–	–	–	[6.5–12.5]

**Table 3:** Values of electrophysiological parameters used for selecting models in the control population.

**Metric**	**Calibration criteria**	**All 3000 models**		**Calibrated**	
		**Mean**	**Std**	**Mean**	**Std**
APD_90_ (ms)	80–120	165	126	96	13
APD_40_ (ms)	20–60	98	91	57	6
APA (mV)	90–140	102	31	121	7
RMP (mV)	< −75	−57	37	−78	2
CaT-A (μM)	0.1–1.0	1.0	2.9	0.30	0.05
[Na^+^]_i_ (mM)	8–12	11.7	6.4	9.5	2.0
CaT rise time	<100 ms	89	60	97	11
EAD	Absent	38%		Absent	
Alternans	Absent	18%		Absent	

*(Values were based on experimental data in Table 2), and corresponding mean and standard deviation (std) values of the original and control populations*.

### 2.4. Analysis of Calcium Wave Propagation

The primary focus of this paper is on Ca^2+^ dynamics, and the Ca^2+^ signal and wave dynamics were subject to a more extensive analysis than the other output variables. Spatio-temporal plots of Ca^2+^ dynamics, resembling line-scan plots of Ca^2+^ fluorescence, were created to asses the radial propagation of Ca^2+^. More detailed analysis of spatial variations in Ca^2+^ signal morphology was done by comparing plots of the cytosolic CaTs (Ca_*i*_) in the membrane domains (domains #1 and #18), and in the central domains (domains #9 and #10). The membrane and central CaTs are denoted as CaT_m_ and CaT_c_, respectively. When using deterministic RyR formulations, the model is symmetric about the cell center, resulting in identical CaTs in domains 1 and 18, as well as in domains 9 and 10. Whole-cell CaTs were also obtained as the mean bulk cytosolic CaTs of the 18 domains.

**Figure 3:**
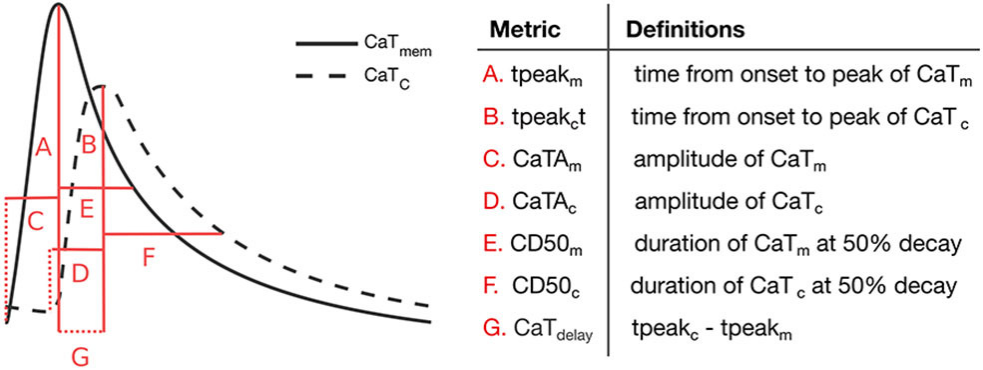
Ca^2+^ transient metrics defined to compare peripheral and central CaT traces, and corresponding descriptions.

Additionally, we extracted and analyzed a number of relevant metrics that characterize CaT_m_ and CaT_c_ traces at steady state (20 min pacing). Specifically, we analyzed the rise time (measured from onset of the CaT to peak), duration at 50% decay (CD50), and amplitude, in addition to the time difference between peaks of CaT_c_ and CaT_m_, as measured from AP onset (CaT_delay_), represented schematically in Figure 3. The Ca^2+^ metrics presented were again determined as the average of the values calculated for the last 5 beats.

We additionally extracted Ca_*SR*_ and [Na^+^]_i_ traces to analyse in relation to the other CaT metrics. In our analyses, we determined Ca_*SR*_ and [Na^+^]_i_ as the mean value during each beat, with Ca_*SR*_ corresponding to the average over all cell domains.

Finally, we performed correlation analysis to identify significant correlations between selected model parameters and output variables. We analyzed the correlations between the maximum ion channel conductances and the Ca^2+^ metrics defined above, as well as correlations between the Ca^2+^ metrics in the membrane domains and in the central domains, and between Ca_*SR*_ and [Na^+^]_i_ and the central Ca^2+^ metrics. The correlations were determined using Kendall's tau (τ), since the variables followed discrete distributions. Significance was determined with 95% confidence, and all data analyses were performed in Matlab R2017a employing custom routines.

## 3. Results

### 3.1. Population of Models

Varying the maximum conductances between 25 and 400% resulted in an initial population of 3,000 models with a large degree of variation in AP and CaT properties. Calibration of the population by constraining output values to the ranges in Table 3 resulted in the selection of 16 out of 3,000 models. Restricting values of APD_90_, APD_40_, and CaT-A was responsible for excluding the majority of models, with 175 models satisfying the requirements for these three parameters. Mean and standard deviation of APD_90_, APD_40_, APA, RMP, CaT-A, [Na^+^]_i_, and CaT rise time for the original and control populations are shown in Table 3. EAD and alternans in the entire population are shown as % of occurrence.

**Figure 4:**
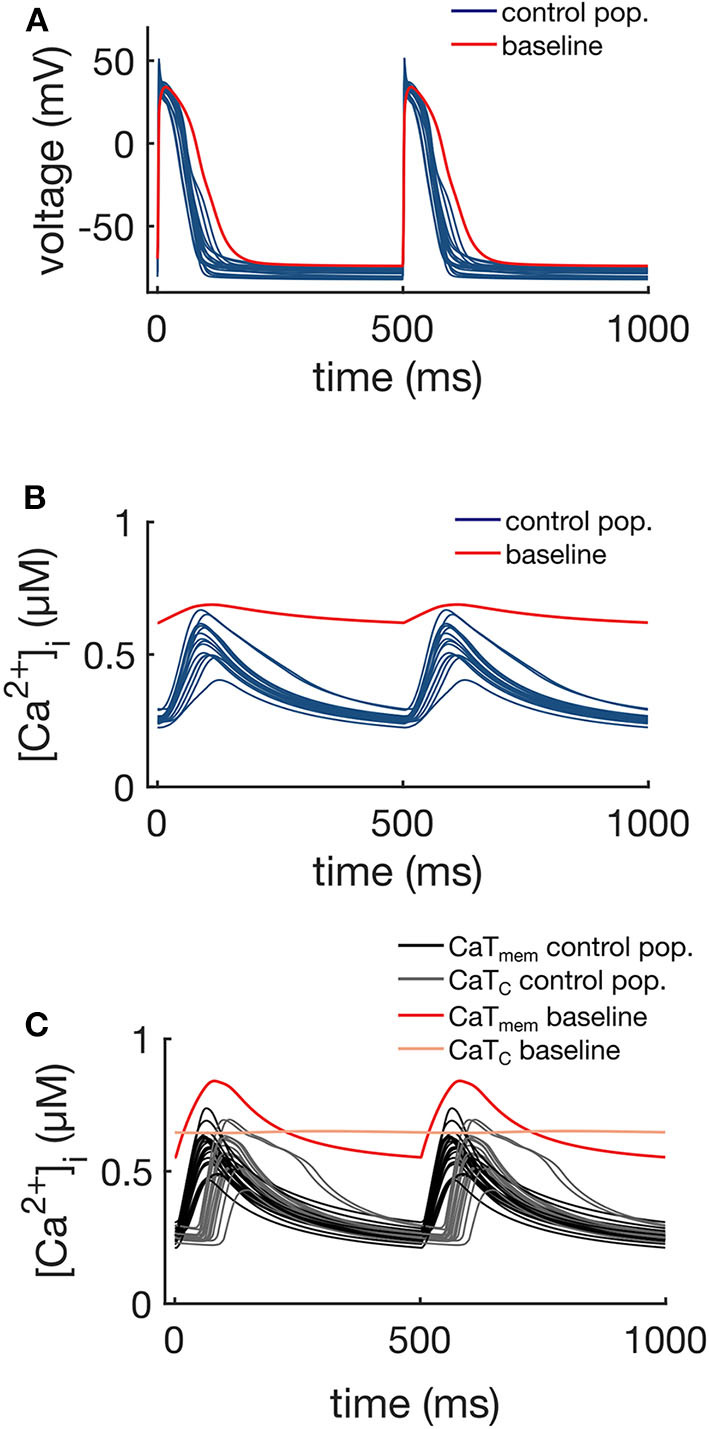
APs **(A)**, whole-cell CaTs **(B)**, and membrane (CaT_m_) and center of cell (CaT_c_) CaTs **(C)** of the control population (16 models), at 2 Hz steady pacing. The red traces represent the baseline model with the original maximum conductances shown in Table 1.

**Figure 5:**
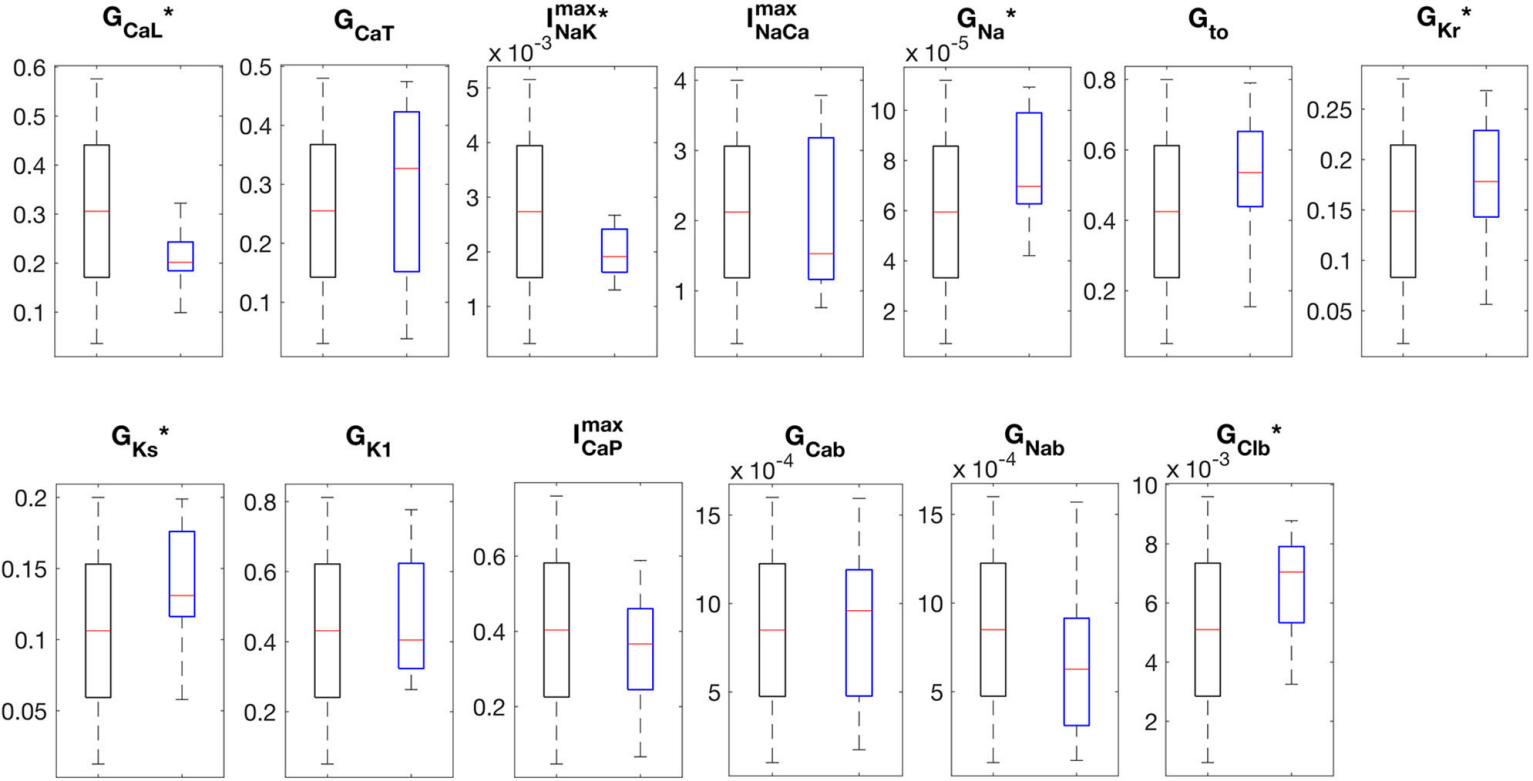
Boxplots of the distributions of maximum conductances. The “*” indicates distributions that were significantly different between the original (black boxes, *n* = 3000) and control populations (blue boxes, *n* = 16) (Kolmogorov-Smirnov test, *p*-val < 0.05).

The APs and CaTs of the control population are shown in Figures 4A,B. Although the model selection step obviously reduced the variability in APs and CaTs, the maximum values of the ionic conductances retained a relatively large range of variation. Only six of the thirteen varied maximum conductances showed significantly reduced variation in the control as compared to the whole population (G_**CaL**_, ${\text{I}}_{\text{NaK}}^{max}$, G_Na_, G_Kr_, G_Ks_, and G_Clb_). The reduced variability in these six parameters was found to be significant (two-sample Kolmogorov-Smirnov test, α = 0.05), and the distributions are illustrated in Figure 5. This indicates that these ion currents affect the rabbit AP and CaT morphology to a larger extent than the other conductances, and also that these parameters are easier to constrain and identify using the considered metrics.

Figure 4C shows the CaT_m_ and CaT_c_ traces of the control population. As expected, the Ca^2+^ signals at these two different locations showed differences in morphology, resulting from the differences in the underlying mechanisms driving the signal. The membrane signal CaT_m_ results from a combination of Ca^2+^ entering the cell via I_CaL_ and Ca^2+^ released from junctional CRUs, while the central signal CaT_c_ is the Ca^2+^ released from non-junctional CRUs via CICR as a result of Ca^2+^ diffusing from neighboring domains. The CaT_c_ is therefore initiated with a time delay in comparison to CaT_m_, which corresponds to the diffusion time of Ca^2+^ from the periphery to the central regions of the cell.

### 3.2. Calcium Dynamics and Wave Propagation

The values of the Ca^2+^ metrics for 16 models are shown in Table 4. Both the rise time and CD50 were significantly different between the CaT_m_ and CaT_c_ traces (two-sample Kolmogorov-Smirnov test, α = 0.05), with no significant differences in the amplitudes of CaT_m_ and CaT_c_, which is consistent with findings in control atrial CMs (Greiser et al., 2014). The CaT_c_ showed a shorter tpeak (time to peak from central AP onset) and a longer CD50 than CaT_m_. Furthermore, CaT_delay_ in the population was 42 ± 12 ms, which matches the 52 ms time delay measured from line scans of rabbit atrial CMs (Greiser et al., 2014). While Ca_*SR*_ was fairly consistent across the 16 models (0.30 μM), [Na^+^]_i_ showed a large degree of variation among models.

**Table 4:** Values of the Ca^2+^ metrics obtained from the control population.

**Model**	**tpeak_m_** **(ms)**	**tpeak_c_** **(ms)**	**CaT_delay_** **(ms)**	**CaTA_m_** **(μM)**	**CaTA_c_** **(μM)**	**CD50_m_** **(ms)**	**CD50_c_** **(ms)**	**[Na^**+**^]_i_**	**Ca_SR_** **(μM)**
1	74	67	47	0.29	0.28	92	96	7.8	0.29
2	88	67	40	0.24	0.28	110	96	7.8	0.29
3	74	61	34	0.32	0.37	103	99	7.5	0.31
4	60	62	41	0.37	0.34	84	98	12.5	0.30
5	72	63	38	0.35	0.32	92	98	6.9	0.30
6	78	62	34	0.27	0.41	133	166	10.6	0.32
7	63	67	51	0.39	0.27	71	96	10.9	0.29
8	71	61	31	0.35	0.37	99	99	10.5	0.31
9	72	67	50	0.29	0.28	86	96	8.9	0.29
10	70	64	38	0.30	0.31	95	98	8.2	0.30
11	67	63	38	0.39	0.32	85	99	11.1	0.30
12	65	61	36	0.50	0.36	73	99	10.4	0.31
13	89	67	48	0.25	0.27	108	96	7.6	0.28
14	70	74	79	0.25	0.21	95	94	7.1	0.26
15	65	63	36	0.41	0.41	99	192	12.3	0.32
16	68	61	31	0.39	0.37	84	99	12.5	0.31
Mean	72	64	42	0.33	0.32	94	109	9.5	0.30
Std	8	4	12	0.07	0.06	15	28	2.0	0.02

**Figure 6:**
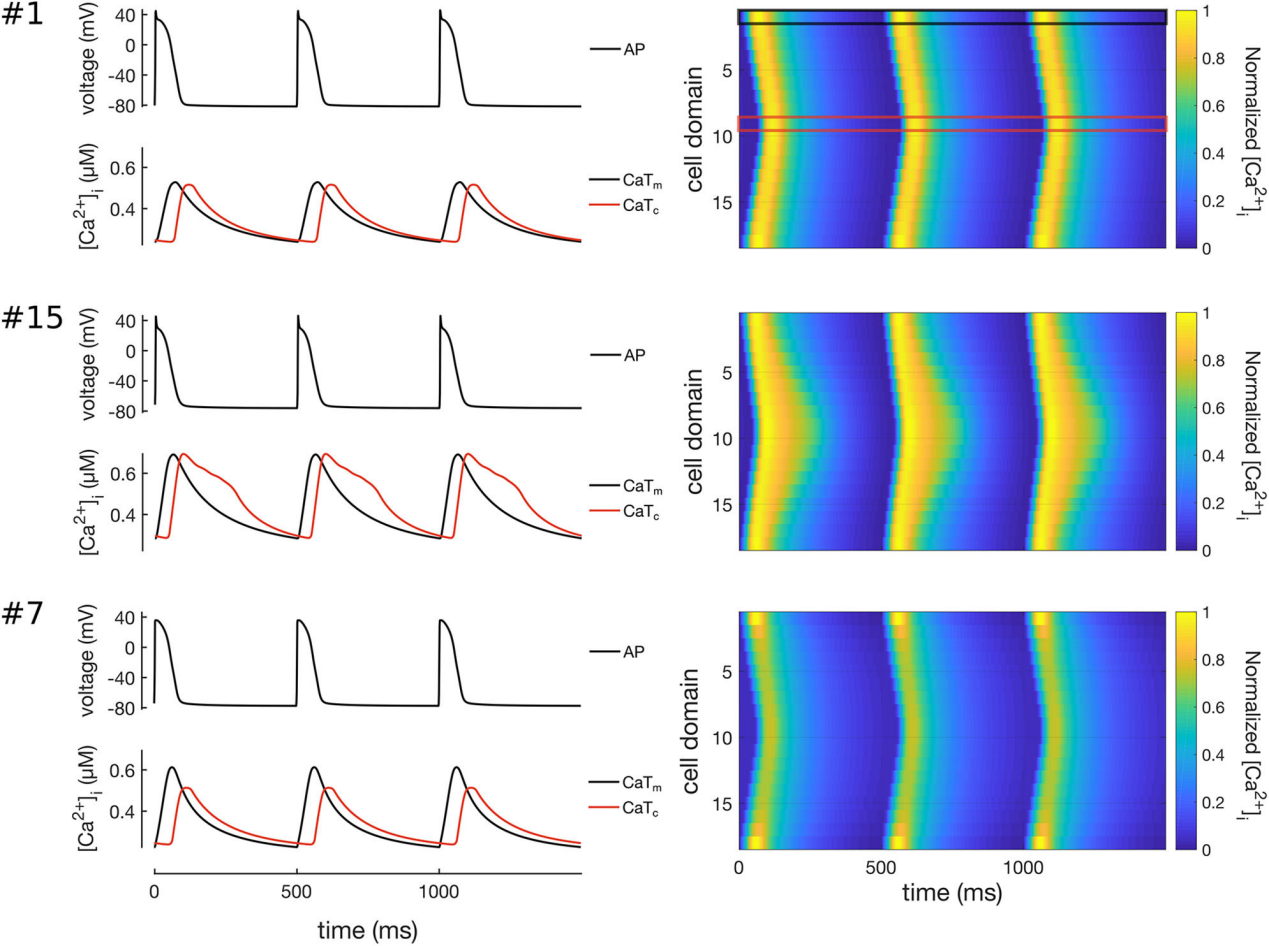
Simulation of Ca^2+^ waves in three representative models of the control population (models #1, #7, and #15). Traces on left hand-side show the APs (top), CaTs (bottom) at the membrane (CaT_m_, black lines) and centre of cell (CaT_c_, red lines). The right hand-side of the Figure shows line scans of propagating Ca^2+^ waves in 3 simulated beats at 2 Hz pacing. The black and red boxes on the top-right panel show the domains corresponding to the CaT_m_ and CaT_c_ traces. Model #1 shows a propagating Ca^2+^ wave where CaT_m_ and CaT_c_ have rather similar morphologies; model #15 shows a Ca^2+^ wave with slightly larger CaT_delay_; and model #7 shows a wave patterns with decreased CaTA_c_ as compared to CaTA_m_.

All 16 models of the control population showed similar Ca^2+^ wave propagation patterns with physiological characteristics under normal conditions, including full regenerative propagation, delayed CaT_c_, and steady CaTs over time. However, despite the similarities there were also measurable differences in the propagation patterns, as characterized by the Ca^2+^ metrics defined above and by plots of the spatio-temporal Ca^2+^ dynamics. Three representative Ca^2+^ wave propagation patterns (models 1, 15, and 7 in Table 4) are also shown in Figure 6. Model #1 corresponds to a CaT_delay_ very close to the average, and with similar CaTA_m_ and CaTA_c_; model #15 shows a shorter CaT_delay_, and slowed CaT_c_ decay; and model #7, in turn, showed a slightly longer CaT_delay_, but a damping of CaT_c_.

We also noted that models #6 and #15 showed a biphasic CaT_c_ decay, with a distinct Ca^2+^-wave pattern as compared to all the other models in the control population. The most likely candidate mechanism is a reduced Ca^2+^-extrusion rate, which causes cytosolic Ca^2+^ to accumulate in the inner domains.To investigate this mechanism we first compared the maximum conductances of I_CaL_, I_CaP_, I_NCX_, and I_NaK_ in the two models. We noticed that the models corresponded to two distinct combinations of the maximum conductances of these four ionic currents, which suggests that the mechanisms underlying the prolonged CaT_c_ might be different.

Model #6 showed a combination of reduced peak magnitude of I_CaL_, I_NaK_, I_NCX_, and slightly reduced peak I_CaP_ (despite a large ${\text{I}}_{\text{CaP}}^{max}$). In contrast, model #15 showed reduced peak I_NaK_ and I_CaP_, only slightly reduced I_NCX_, and increased I_CaL_. Thus, the common characteristic in the two models was a low I_NaK_ magnitude. An interesting observation is that in model #6 the magnitude of I_NCX_ was reduced, yet to a lesser extent than ${\text{I}}_{\text{NCX}}^{max}$, which means that in this model the increased [Ca^2+^]_i_ still resulted in an enhancement of I_NCX_. A possible mechanism in model #6 may be simply the reduced ${\text{I}}_{\text{NCX}}^{max}$ which resulted in [Ca^2+^]_i_ accumulation and the observed prolongation of CaT_c_. In model #15, however, the mechanism seems to be different, possibly the reduced I_NaK_ resulted in an accumulation of [Na^+^]_i_, which further reduced the activity of I_NCX_, and consequently increased [Ca^2+^]_i_ prolonging CaT_c_. This was paralleled by a smaller extrusion via I_CaP_.

**Figure 7:**
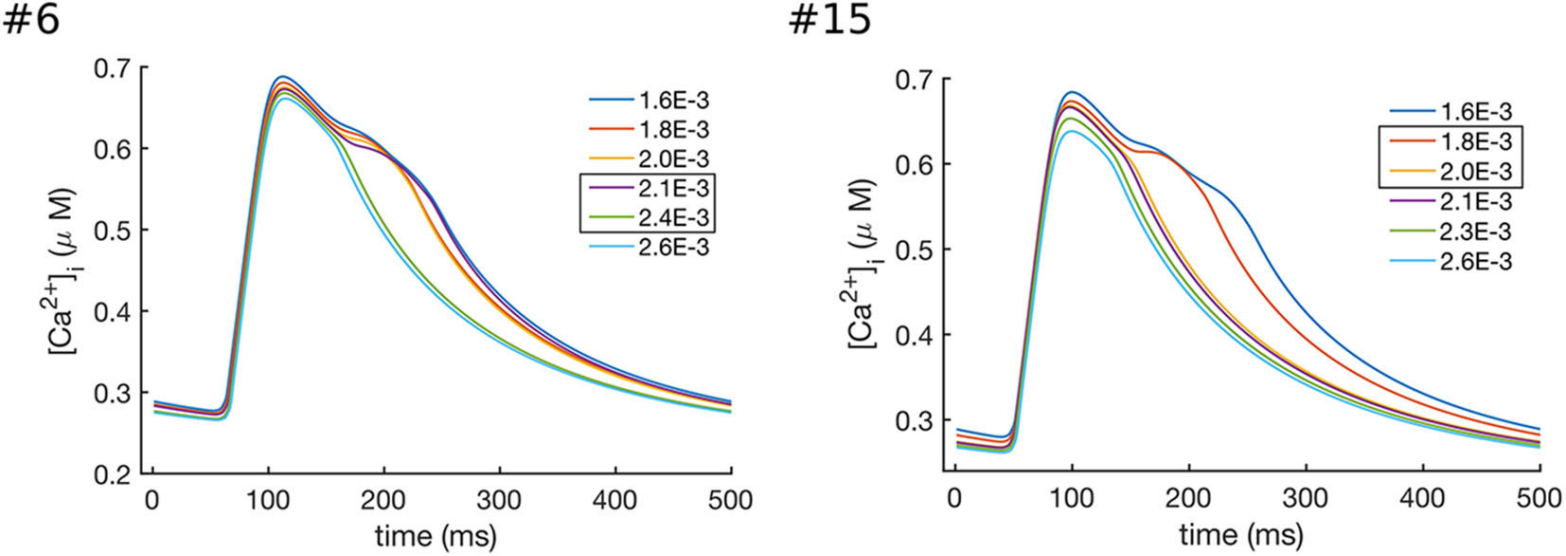
Effects of increasing the values of ${\text{I}}_{\text{NaK}}^{max}$ in models #6 and #15, which resulted in the enhanced Ca^2+^ extrusion and a faster CaT_c_ decay. In model #6 the transition between the biphasic and smooth CaT profile occurred at values of ${\text{I}}_{\text{NaK}}^{max}$ between 2.1 × 10^−3^ and 2.4 × 10^−3^ ns/pF, while in model #15 the transition occurred at values between 1.8 × 10^−3^ and 2.0 × 10^−3^ ns/pF.

**Figure 8:**
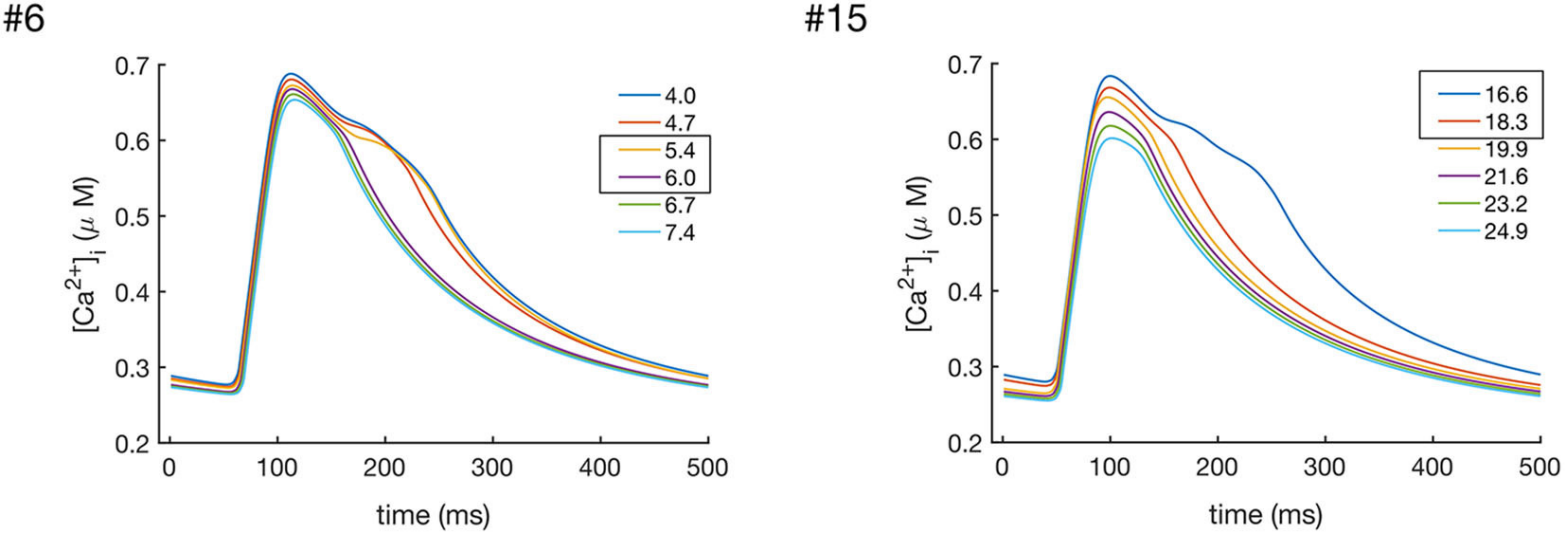
Effects of increasing the values of ${\text{I}}_{\text{NCX}}^{max}$ in models #6 and #15, which resulted in the enhanced Ca^2+^ extrusion and a faster CaT_c_ decay. In model #6 the transition between the biphasic and smooth CaT profile occurredat values of ${\text{I}}_{\text{NCX}}^{max}$ between 5.4 and 6.0 nS/pF, while in model #15 the transition occurred at values between 16.6 and 18.3 nS/pF.

In order to investigate the role of I_NaK_ and I_NCX_ in the decay of CaT_c_, we gradually increased the values of ${\text{I}}_{\text{NaK}}^{max}$ and ${\text{I}}_{\text{NCX}}^{max}$ individually. The results of these simulations are shown in Figures 7, 8. As can been seen from these results, increasing either ${\text{I}}_{\text{NaK}}^{max}$ or ${\text{I}}_{\text{NCX}}^{max}$ decreased the CaT_c_ decay time, abolishing the biphasic decay behavior, although at different thresholds for each of the models. In model #6 the threshold for transition from biphasic to monotonic decay occurred at 80% increase of ${\text{I}}_{\text{NaK}}^{max}$ and 120% increase of ${\text{I}}_{\text{NCX}}^{max}$, whereas in model #15 the transition occurred at 82 and 54% increase of ${\text{I}}_{\text{NaK}}^{max}$ and ${\text{I}}_{\text{NCX}}^{max}$, respectively. Although these results do not allow to discern the relative contribution of I_NCX_ and I_NaK_ to the prolongation and biphasic behavior of CaT_c_ decay due to the small number of observations, they do seem to indicate that these two currents play a major part in determining the decay of CaT_c_, as is expected given their role in regulating [Ca^2+^]_i_.

### 3.3. Correlation Analysis

We next analyzed the correlations between the maximum conductances of individual currents and the Ca^2+^ metrics defined in Figure 3. The results are summarized in Table 5, where significant correlation coefficients (*p*-value < 0.05) are highlighted in bold. Our analyses reveal that the Ca^2+^ wave properties of these 16 models were primarily sensitive to G_**CaL**_, ${\text{I}}_{\text{NCX}}^{max}$, and ${\text{I}}_{\text{NaK}}^{max}$. The L-type Ca^2+^ channel conductance G_**CaL**_ showed a negative correlation with CD50_m_, but not with CaTA_m_ and CaTA_c_. ${\text{I}}_{\text{NCX}}^{max}$ was negatively correlated with tpeak_m_ and CD50_m_, which is the expected given role of I_NCX_ in Ca^2+^ extrusion. ${\text{I}}_{\text{NCX}}^{max}$ was also positively correlated with CaTA_m_, possibly because larger CaTs increase NCX activity. However, ${\text{I}}_{\text{NCX}}^{max}$ did not affect CaT_delay_. Finally, we found ${\text{I}}_{\text{NaK}}^{max}$ to be negatively correlated with CaTA_c_ and CD50_c_, indicating that a larger I_NaK_ current resulted in a smaller CaT_c_ signal.

**Table 5:** Kendall's τ and corresponding *p*-values of the correlations between maximum conductances and CaT_m_ and CaT_c_ properties.

**Param**.	**tpeak**_****m****_		**tpeak**_****c****_		**CaT**_****delay****_		**CaTA**_****m****_		**CaTA**_****c****_		**CD50**_****m****_		**CD50**_****c****_	
	**τ**	***p*-val**	**τ**	***p*-val**	**τ**	***p*-val**	**τ**	***p*-val**	**τ**	***p*-val**	**τ**	***p*-val**	**τ**	***p*-val**
G_**CaL**_	−0.33	0.086	0.13	0.52	0.31	0.11	0.30	0.12	−0.23	0.23	−0.51	0.0076	−0.21	0.31
G_CaT_	0.093	0.65	0.13	0.52	0.10	0.62	−0.25	0.19	−0.18	0.35	0.10	0.62	−0.25	0.23
${\text{I}}_{\text{NaK}}^{max}$	0.14	0.47	0.27	0.17	0.31	0.11	−0.20	0.31	−0.43	0.020	−0.14	0.50	−0.54	0.007
${\text{I}}_{\text{NCX}}^{max}$	−0.72	0.0001	−0.079	0.71	0.10	0.62	0.45	0.015	−0.050	0.82	−0.75	0.0001	−0.045	0.85
G_Na_	−0.19	0.32	−0.044	0.85	−0.051	0.82	0.22	0.27	0.017	0.97	−0.15	0.44	0.082	0.71
G_to_	−0.11	0.59	0.13	0.52	0.10	0.62	0.10	0.63	−0.13	0.51	−0.12	0.56	−0.082	0.71
G_Kr_	−0.16	0.42	−0.11	0.58	0.017	0.96	0.13	0.51	−0.033	0.89	−0.22	0.26	−0.064	0.78
G_Ks_	−0.21	0.28	0.13	0.52	0.19	0.34	0.0	1.0	−0.033	0.89	−0.17	0.39	−0.064	0.78
G_K1_	−0.36	0.058	0.062	0.78	0.17	0.39	0.15	0.45	−0.083	0.69	−0.27	0.16	−0.12	0.58
G_CaP_	0.30	0.12	−0.027	0.93	−0.068	0.75	−0.30	0.12	0.067	0.76	0.27	0.16	−0.10	0.64
G_Cab_	−0.13	0.53	−0.22	0.27	−0.14	0.50	0.10	0.63	0.10	0.63	−0.24	0.22	0.064	0.78
G_Nab_	0.093	0.65	−0.34	0.082	−0.31	0.11	−0.083	0.69	0.15	0.45	−0.017	0.96	0.12	0.58
G_Clb_	−0.30	0.12	0.20	0.31	0.29	0.13	0.10	0.63	−0.20	0.31	−0.085	0.68	−0.17	0.40

The correlation among the seven Ca^2+^ metrics, [Na^+^]_i_, and Ca_*SR*_ are compiled in Table 6, with significant correlation coefficients (*p*-value < 0.05) highlighted in bold. We observe that CaT_delay_ which was not sensitive to any of the ion current conductances, was strongly correlated with Ca_*SR*_. We also found [Na^+^]_i_ to be correlated with tpeak_m_, CaTA_m_, and CD50_c_. Furthermore, we observe that the individual Ca^2+^ metrics related to CaT_m_ and CaT_c_ were in general not correlated, except for CaTA_m_ which was correlated with tpeak_c_, and CD50_c_, while CaT_delay_ was strongly correlated with all CaT_c_ properties, tpeak_c_, CaTA_c_, and CD50_c_. The three Ca^2+^ metrics t_*rise*_, CD50, and amplitude, were correlated for both CaT_m_ and CaT_c_, as expected (data not shown).

**Table 6:** Kendall's τ and corresponding *p*-values of the correlations between CaT_m_ and CaT_c_ properties, [Na^+^]_i_, and Ca_*SR*_.

**Parameters**	**tpeak**_****c****_		**CaTA**_****c****_		**CD50**_****c****_		**CaT**_****delay****_		**[Na**^****+****^**]**_****i****_		**Ca**_*******SR*******_	
	**τ**	***p*-val**	**τ**	***p*-val**	**τ**	***p*-val**	**τ**	***p*-val**	**τ**	***p*-val**	**τ**	***p*-val**
tpeak_m_	0.18	0.38	−0.093	0.65	−0.15	0.49	0.0	1.0	−0.44	0.019	−0.093	0.65
CaTA_m_	−0.41	0.035	0.33	0.08	0.43	0.03	−0.27	0.16	0.40	0.033	0.33	0.079
CD50_m_	0.081	0.71	0.10	0.62	0.10	0.64	−0.16	0.44	−0.25	0.19	0.10	0.62
CaT_delay_	0.79	0.0001	−0.77	0.0001	−0.77	0.0001	1	0	−0.26	0.19	−0.77	0.0001
[Na^+^]_i_	−0.34	0.082	0.37	0.052	0.41	0.041	−0.26	0.19	1	0	0.37	0.052
Ca_*SR*_	−0.79	0.0001	1.0	0.0	0.88	0.00	−0.77	0.0001	0.37	0.052	1	0

## 4. Discussion

We have developed a model of a healthy atrial CM with rabbit-specific EP and spatially distributed Ca^2+^ dynamics. The central motivation for developing the model was to be able to describe radial diffusion of calcium, which is important for the investigation of the effects of asynchronous Ca^2+^ release on arrhythmic activity in atrial CMs lacking TTs. We used a population-of-models approach to parameterize the maximum conductances of sarcolemmal ion currents to produce a pool of models that matched reported experimental data from the rabbit atria. The result was a population of 16 models that were all consistent with observed experimental values, but still recapitulated observed variability in Ca^2+^ wave characteristics.

The small number of models selected by the experimental data shows that imposing a sufficiently large number of constraints in model outputs (in this case, eight parameters) can reduce the parameter space to discrete sets of parameterizations, each following a unique trajectory. This shows that rather different parameter combinations can result in models with very similar behaviors (see Figure 4 and Supplementary Table 3, highlighting the non-uniqueness of CM models, a consequence of the compensatory effects of ionic currents (Sarkar and Sobie, 2010; Zaniboni, 2011; Muszkiewicz et al., 2018). Nonetheless, the model selection step significantly reduced the variability in six of the maximum conductances, as seen in Figure 5, indicating that the electrophysiology and Ca^2+^ metrics used in the model selection step were, in general, sensitive to these maximum conductances.

It is worth noting that the data used here to constrain the model were obtained from a large assortment of published experimental data. Therefore, the 16 models of the control population reflect not only the natural variability observed within different atrial regions, but also experimental uncertainties inherent to methodologies used by different research groups. The population-of-models-based approach we used here contrasts with the more standard approach, wherein a computational model is fitted to a small set of experimental observations, often obtained from a single atrial region, to yield a single model parameterization that captures the average behavior in the experimental data. Although useful for assessing the mechanisms underlying general characteristic behaviors of the model, the single-parameter approach lacks the ability to reproduce experimental observations from a range of data. In contrast, incorporating variability into the model via the population-of-models-based approach employed here allows a generalization of model results to a wider set of conditions and phenotypes.

### 4.1. Correlations Analysis

The seven extracted Ca^2+^ metrics from CaT_m_ and CaT_c_ quantify differences in the Ca^2+^ wave properties across the 16 models. Correlation analysis showed that G_**CaL**_ and ${\text{I}}_{\text{NCX}}^{max}$ and ${\text{I}}_{\text{NaK}}^{max}$ were the only maximum conductances significantly affecting Ca^2+^ wave propagation in the model. This result is consistent with the known role of these ionic currents in intracellular Ca^2+^ regulation. For instance, experimental observations have shown the role of increased sarcolemmal Ca^2+^ in modulating the regenerative propagation of the Ca^2+^ signal toward the inner locations of the cell (Mackenzie et al., 2004).

The strong correlation between ${\text{I}}_{\text{NCX}}^{max}$ and CaT_m_ metrics indicates a strong modulating effect of I_NCX_ on Ca^2+^ dynamics at the cell membrane. The importance of the role of I_NCX_ on modulation of Ca^2+^ dynamics during the AP is well-documented both experimentally, and through computational simulations (Hilgemann et al., 1992; Sher et al., 2008; Xie et al., 2015). Furthermore, since I_NaK_ affects [Na^+^]_i_ homeostasis, which in turn affects I_NCX_ function, it is not surprising that I_NaK_ was correlated with CaTA_c_ and CD50_c_. However, it is somewhat unexpected that I_NaK_ was more strongly correlated to the CaT_c_ than to CaT_m_ properties.

The observed correlations between CaTA_m_ and tpeak_c_ and CD50_c_ indicates that the rate of Ca^2+^ release and uptake in the CRUs is modulated to some extent by the amount of Ca^2+^ that enters the membrane and initiates CICR. We also observed that CaT_delay_ was correlated to tpeak_c_, CaTA_c_, and CD50_c_, which indicates that the velocity of Ca^2+^ wave propagation was mostly modulated by the dynamics of the regenerative propagation of the Ca^2+^ signal along CRUs, and not by the amount of Ca^2+^ entering via the cell membrane. CaT_delay_ measures the time for the Ca^2+^ wave to propagate to the innermost cell domains which depends on the strength and rate of the regenerative CICR. This also determines the shape of the local CaTs in each inner domains. Therefore, it is expected that CaT_delay_ co-varies with the CaT_c_ properties, but not with the CaT_m_ properties.

The observation that CaT_delay_ strongly correlated with Ca_*SR*_ is not unexpected, since a higher SR Ca^2+^ load would naturally promote a faster rise of the CaT at inner domains (smaller tpeak_c_), and thus reduce CaT_delay_, while simultaneously promoting a longer CaT duration (larger CD50_c_). The observed correlation between Ca^2+^ metrics and [Na^+^]_i_ is also expected since [Na^+^]_i_ plays a significant role in the modulation of NCX function, and therefore in the regulation of the sub-sarcolemmal Ca^2+^ signal (Hilgemann et al., 1992; Sher et al., 2008; Xie et al., 2015).

Overall the results of the correlation analyses presented here are in good agreement with our understanding of Ca^2+^ handling dynamics in atrial CMs, and provide additional insight into the mechanisms driving Ca^2+^ wave propagation in the newly developed model taking into account population variability.

### 4.2. Limitations and Future Directions

It is important to note that the conclusions derived from the analyses presented here are limited by the experimental data that was used in the model selection step. In particular, the lack of quantitative measurements of intracellular Ca^2+^ makes it difficult to validate the model predictions of the spatial characteristics of the Ca^2+^ dynamics. The correlation analysis used in this paper also has its own limitations and assumptions. For instance, simultaneously varying parameters to build the population can lead to interaction effects, which can mask the individual contributions of each varied parameter. The analyses presented here can be extended by, for example, determining multivariate correlations. This would require increasing the sample size to allow for a more robust regression analysis of the parameters. A way to achieve this would be by constructing new models by perturbing the parameters around the values that originated the 16 models. Alternatively, partial correlations can be determined by removing the effect of collinearity of variables.

Furthermore, the developed pool of normal rabbit atrial CM models can be used to study the effects of altered Ca^2+^ handling parameters, such as Ca^2+^ release and uptake from the SR, and Ca^2+^ buffering strength on Ca^2+^ wave propagation dynamics. Variability in these parameters will likely have a stronger modulating effect on the Ca^2+^ wave metrics studied here than the ionic currents, and thus constitute a natural extension of the present work.

Another relevant model limitation is the use of a deterministic RyR model, which implicitly assumes homogeneous distribution and behavior of CRUs. Therefore, this simplification does not include stochastic Ca^2+^ release events and subcellular fluctuations in calcium-handling proteins, which may influence the results (Sutanto et al., 2018) and limit the applicability of the model to describe pathological conditions. Using a stochastic formulation would allow the incorporation of spatial heterogeneity in Ca^2+^ release, which constitutes an important trigger for spontaneous Ca^2+^ waves, and consequently for abnormal Ca^2+^-wave propagation patterns. The current model implementation has limited ability to describe these events, but it can easily be extended to a stochastic RyR model to address the role of Ca^2+^ release stochasticity in Ca^2+^ wave dynamics. Additionally, the model can easily be extended to incorporate additional layers of intra- and inter-cellular variability, including TT structures.

We also recognize that the Ca^2+^-handling model from the Voigt et al. (2014) has the intrinsic limitations of not being specific to spatial Ca^2+^ distribution in rabbit atrial CMs. Previously developed atrial CM models with rabbit-specific Ca^2+^ handling include the models from Hilgemann and Noble (1987) and from Lindblad et al. (1996). Hilgemann and Noble (1987) proposed a 4-state model of the Serca pump with a component dependent on SR Ca^2+^ to incorporate Serca regulation by luminal Ca^2+^. Lindblad et al. (1996) modified this formulation to take into account additional experimental observations, Ca^2+^ buffering, and to adjust graded Ca^2+^ release and CICR in the model. The Shannon rabbit ventricular CM model (and our model), in turn, uses a bi-directional Hill equation with affinity for cytosolic and SR Ca^2+^, although the formulation used here (from the Voigt & Heijman model) had been adjusted to human atrial CM data. Additionally, Ca^2+^-release in Hilgemann and Noble (1987) is modeled as a simple function of SR Ca^2+^ concentration, while in Lindblad et al. (1996) this formulation was modified to also take into account cytosolic Ca^2+^. On the other hand, the Shannon model and our model use a 4-state Markov Model that allows to simulate local control of Ca^2+^ release. Hilgemann and Noble (1987) and Lindblad et al. (1996) used essentially the same formulation for the Na^+^-Ca^2+^ exchanger, while both the Shannon and our model use a similar formulation for I_NCX_ with additional terms to reflect Na^+^ and Ca^2+^ dependence. Thus, given the differences in Ca^2+^ handling formulations in the various models, it is fair to say that there is considerable uncertainty regarding which model structure is the most appropriate, especially also because of the limited amount of data on Ca^2+^ handling in rabbit atrial CMs. Future work should investigate how these different Ca^2+^ models reflect overall Ca^2+^ dynamics in rabbit atrial CMs by comparing Ca^2+^ wave propagation patterns. We note that, to the best of our knowledge, this study is the first attempt at providing a systematic approach for analyzing intracellular Ca^2+^ wave propagation in a rabbit atrial cardiomyocyte model with spatial Ca^2+^ handling.

We also note that RyR density in the model should be adjusted to reflect the smaller size of rabbit as compared to human atrial CMs. The model assumes a transversal spacing of 0.7 μm, which is smaller than the 1 μm spacing assumed in the original Voigt & Heijman model. This is an important consideration since the number of RyR has a direct impact on the amplitude of the cytosolic Ca^2+^ signal and on Ca^2+^ diffusion, and consequently on Ca^2+^ wave propagation dynamics. An overestimation of these parameters may result in exacerbated Ca^2+^ propagation which does not necessarily reflect physiological Ca^2+^ diffusion. Nevertheless, the model can also be simulated with fewer domains. Thus, further developments of the model should address this by adjusting RyR density and Ca^2+^ diffusion parameters, and Ca^2+^ diffusion properties should be validated against rabbit data, for example as done in Sutanto et al. (2018).

Another important consideration is the effect of TTs on atrial CM Ca^2+^ cycling. Although a general absence of significant TT networks has been reported in rabbit atrial CMs (Tidball et al., 1991; Blatter, 2017), a large regional variability in TT density across the atria, as well as the presence of axial TTs has been observed in various species, with corresponding variability in Ca^2+^ release synchrony profiles (Richards et al., 2011; Frisk et al., 2014; Brandenburg et al., 2018). Thus, the implications of considering TT networks in atrial myocytes should be discussed in more detail here. Several computational studies have explored the role of TTs in modulating intracellular Ca^2+^ propagation and disturbances. For example, Nivala et al. (2015) studied the effects of spatial heterogeneity in LTCC distribution and [Na^+^]_i_ on Ca^2+^ alternans propensity in a model with three-dimensional diffusion. Their results showed that TT disruption did not cause Ca^2+^ alternans by itself but promoted their occurrence and lowered the onset threshold when acting in concert with increased [Na^+^]_i_ (which increased Ca^2+^ load), and down-regulation of Serca (together with increased [Na^+^]_i_), with alternans amplitude increasing with increasing percentage of TT disruption or [Na^+^]_i_. This study emphasized not only the role of the spatial distribution of LTCCs in the generation of alternans, and thus the importance of considering spatial heterogeneity of Ca^2+^ cycling, but also the role of regulating [Na^+^]_i_ on abnormal intracellular Ca^2+^ propagation.

Colman et al. (2017) performed a similar study on a model with 3D spatial Ca^2+^ cycling with variable TT density, showing that decreased TT density correlated with increased propensity for Ca^2+^ alternans. Their simulations with patchy TTs also showed observable spatial Ca^2+^ gradients leading to the formation of intracellular Ca^2+^ waves at the sites with no TTs and of delayed after depolarizations. In a similar study, Song et al. (2018) also presented a 3D spatial model of Ca^2+^ incorporating different TT architectures, which showed an increased incidence of Ca^2+^ alternans and intracellular Ca^2+^ wave formation in non-uniform random TT networks, as compared to uniform networks.

Marchena and Echebarria (2018) also developed a model of intracellular spatial Ca^2+^ distribution, but using a different framework based on a submicron resolution grid of points, allowing for a more refined description of CM subcellular structure with transverse TTs at the z-lines. At the membrane level, RyR and LTCC clusters are distributed at regular intervals, whereas inside the CM the RyR clusters follow a Gaussian distribution centered at the z-line, with only some of the grid points containing CRUs. Furthermore, they defined cytosolic, SR, and buffer-bound Ca^2+^ concentrations for each grid. Interestingly, their simulations showed an almost complete absence of Ca^2+^ signal at the center despite their model accounting for TTs. A later study by the same authors (Marchena and Echebarria, 2020) where the same model was used but incorporating variable lengths and distributions of both transverse and axial tubules emphasized the role of these structures on spatial Ca^2+^ propagation, showing a progressive enhancement and synchronization of the Ca^2+^ signal at the cell center with increasing TT density.

Biophysically detailed models of CM ultrastructure with spatially-realistic RyR distribution are also important for understanding structure-function relationships in Ca^2+^ handling. Rajagopal et al. (2015) and Ladd et al. (2019) developed such a model for the rat ventricular CM integrating spatial information from high-resolution imaging that included RyR, myofibrils, and mitochondria, and examined the role of these structures on Ca^2+^ dynamics in a CM cross-section. They showed that incorporating spatially-realistic distribution of RyRs in the model captured the spatial Ca^2+^ heterogeneities observed in line scans. Their simulations also suggest that modeling mitochondria as passive barriers to Ca^2+^ diffusion also introduces heterogeneity in the local CaTs, although to a lesser extent than RyR. These findings further suggest that local distribution of Ca^2+^ re-uptake may also have important consequences for the spatial Ca^2+^ handling, and highlight the importance of considering CM ultrastructure in studying Ca^2+^ diffusion properties.

The effect of adding axial TTs to the Voigt & Heijman human CM model was also studied by Sutanto et al. (2018). Their simulations with variable axial TT density and distribution shows a progressive synchronization of the intracellular Ca^2+^ release in the radial direction. Additionally, their results suggested an increased incidence of spontaneous Ca^2+^ release events when incorporating axial TTs, with the events originating primarily at the RyR clusters adjacent to the axial TTs. Holmes et al. (2018) also presented a model of the rabbit atrial CM implementing the Aslanidi et al. (2009) AP model and the three-dimensional stochastic Ca^2+^ handling model with Ca^2+^ diffusion terms developed by Colman et al. (2017). The latter was based on real geometries and intracellular ultrastructures extracted from sheep ventricular CMs, but otherwise implements a very similar Ca^2+^ handling model as ours and other models. The Holmes et al. (2018) model allowed to simulate the effects of TTs and intracellular Ca^2+^ heterogeneities in spontaneous Ca^2+^ waves. Of note, in their simulations with the fully detubulated model, the CaT morphology was similar to the whole-cell CaT produced by our model, although with a lower diastolic Ca^2+^ level. The CaT delay between the periphery and the center is about 75 ms, ca. 20 ms longer than in our simulations. Although an important result, showing that increased detubulation indeed results in a slowed Ca^2+^ wave propagation, which is in agreement to experimental data, the model was not adjusted to match rabbit specific cellular ultrastructure and intracellular Ca^2+^ diffusion. However, their work does point out the influence of Ca^2+^ dynamics stochasticity on AP shape, although it is not clear from their study how introducing stochasticity in the Ca^2+^ model affected wave propagation. Together these results show that TTs, and in particular axial TTs in atrial CMs, have great importance in modulating intracellular Ca^2+^ propagation and potentially play a role in Ca^2+^ alternans and triggered activity. As such, computational models of atrial CM with spatial Ca^2+^ handling are an essential tool to improve our understanding of Ca^2+^-handling abnormalities in atrial pathophysiology.

Given the wide range of variation of the maximum conductances in the control population (see Figure 5), and the fact that the models were selected entirely based on AP and CaT characteristics, it is natural to ask whether individual ionic currents of the models are in physiological ranges. We have compared simulated I-V curves of I_CaL_, I_Na_, I_to_, I_K1_, and I_Kr_ for the 16 models against experimental data reported by Aslanidi et al. (2009), and Muraki et al. (1995), as well as dynamic current traces from our model population with the Aslanidi model (at steady state at 2 Hz pacing). I-V curves and dynamic current traces are also shown in Supplementary Figures 2, 3, while peak current values are reported in Supplementary Table 4. We observe that the I-V curves are qualitatively similar to the experimental data. Although the range of variation is large, the magnitudes of _CaL_ (−13.9 to −4.3 pA/pF) and _to_ (3.4 to 16.6 pA/pF) are approximately within ±50% of the experimental values (I_CaL_: −7 to −8 pA/pF, I_to_: 8 to 13 pA/pF, Aslanidi et al., 2009). Reported experimental values of peak I_K1_ are between 4 and 5 pA/pF (Aslanidi et al., 2009), whereas the largest peak I_K1_ in our population of 0.6 pA/pF, which is similar to the peak I_K1_ observed in the Aslanidi model, at 0.66 pA/pF. I_Na_, however, was considerably higher in our population (between −737 and −291 pA/pF) as compared to the experimentally reported value of −70 pA/pF. We note, however, that peak dynamic I_Na_ we obtained from the Aslanidi model was −120 pA/pF. Peak I_Kr_ values (0.45–2.1 pA/pF) in our population were also in general considerably larger than the reported experimental value of 0.7 pA/pF (Muraki et al., 1995), assuming a cell capacitance of 50 pF). Peak I_Kr_ in the Aslanidi model was 0.43 pA/pF, which is close to the experimental value.

We also note that variability in experimental recordings of ion currents is typically very large, and ideally this should have been taken into account in the validation of the population. Nevertheless, there are observable discrepancies between peak currents in our control population and in experiments, which are limitations of the model, and we recognize that these should be further investigated. Future iterations in the development of the control population should include fine-tuning of the maximum conductances of the 16 models in order to produce a control population that better matches experimental data.

Finally, a relevant model improvement would be to include the calcium-dependent component of the chloride current, which has been shown to be involved in APD alternans in rabbit atria (Kanaporis and Blatter, 2016), and could therefore potentially affect Ca^2+^ wave propagation dynamics. Future work could also address the effects of AF-induced remodeling on Ca^2+^ wave propagation, and in particular look at the role of RyRs and Serca2a parameters in abnormal behaviors, such as failed Ca^2+^ propagation, Ca^2+^ alternans, and afterdepolarizations.

## 5. Conclusions

This paper presented a novel model of the rabbit atrial CMs, and provides a framework for analysing cardiac cell models based on correlation analysis. We have shown that the model is able to reproduce experimentally observed physiological Ca^2+^ wave propagation patterns. These differences were directly linked to two Ca^2+^ currents, I_CaL_, and I_NCX_. However, the study also showed the Ca^2+^ wave patterns to be a complex interplay among different components, including Ca_*SR*_ and [Na^+^]_i_.

The spatial Ca^2+^ description in the model, along with the methodology presented here can be used as a tool to study sub-cellular mechanisms, and their implication in the arrhythmogenesis in diseased condition, such as atrial fibrillation. This work can therefore be extended to assess such mechanisms under altered conditions, such as electrical remodeling. In particular, the framework can be useful for querying the drivers of arrhythmogenic Ca^2+^ cycling, such as Ca^2+^ alternans, and to formulate hypothesis on new targets to restore normal cell function. In conclusion, the study provides a population of spatial models of the rabbit atrial cardiomyocyte that can serve as a starting point for future studies employing the commonly used experimental model.

## Data Availability Statement

The raw data supporting the conclusions of this article will be made available by the authors, without undue reservation.

## Author Contributions

MV, JH, and US conceived the study. MV performed the computational simulations and data analysis, and drafted the manuscript. HA, JS, and JH provided the critical revision to the manuscript. All authors approved the final version.

## Conflict of Interest

The authors declare that the research was conducted in the absence of any commercial or financial relationships that could be construed as a potential conflict of interest.

## References

[B1] Amrita X. Christini D. J. Sobie E. A. (2012). Exploiting mathematical models to illuminate electrophysiological variability between individuals. J. Physiol. 590, 2555–2567. 10.1113/jphysiol.2011.223313 22495591 PMC3424714

[B2] Aslanidi O. Boyett M. Dobrzynski H. Li J. Zhang H. (2009). Mechanisms of transition from normal to reentrant electrical activity in a model of rabbit atrial tissue: interaction of tissue heterogeneity and anisotropy. Biophys. J. 96, 798–817. 10.1016/j.bpj.2008.09.057 19186122 PMC3325123

[B3] Bers D. M. (2002). Cardiac Na/Ca exchange function in rabbit, mouse and man: what's the difference? J. Mol. Cell. Cardiol. 34, 369–373. 10.1006/jmcc.2002.1530 11991726

[B4] Blatter L. A. (2017). The intricacies of atrial calcium cycling during excitation-contraction coupling. J. Gen. Physiol. 149, 857–865. 10.1085/jgp.201711809 28798277 PMC5583713

[B5] Blatter L. A. Kockskämper J. Sheehan K. A. Zima A. V. Hüser J. Lipsius S. L. (2003). Local calcium gradients during excitation–contraction coupling and alternans in atrial myocytes. J. Physiol. 546, 19–31. 10.1113/jphysiol.2002.025239 12509476 PMC2342467

[B6] Bootman M. D. Berridge M. J. Roderick H. (2002). Calcium signalling: more messengers, more channels, more complexity. Curr. Biol. 12, R563–R565. 10.1016/S0960-9822(02)01055-2 12194839

[B7] Bootman M. D. Smyrnias I. Rädiger Coombes S. Roderick H. L. (2011). Atrial cardiomyocyte calcium signalling. Biochim. Biophys. Acta 1813, 922–934. 10.1016/j.bbamcr.2011.01.030 21295621

[B8] Brandenburg S. Pawlowitz J. Fakuade F. E. Kownatzki-Danger D. Kohl T. Mitronova G. Y.. (2018). Axial tubule junctions activate atrial Ca^2+^ release across species. Front. Physiol. 9:1227. 10.3389/fphys.2018.01227 30349482 PMC6187065

[B9] Chang E. T. Y. Strong M. Clayton R. H. (2015). Bayesian sensitivity analysis of a cardiac cell model using a gaussian process emulator. PLoS ONE 10:e0130252 10.1371/journal.pone.0130252 26114610 PMC4482712

[B10] Colman M. A. Pinali C. Trafford A. W. Zhang H. Kitmitto A. (2017). A computational model of spatio-temporal cardiac intracellular calcium handling with realistic structure and spatial flux distribution from sarcoplasmic reticulum and t-tubule reconstructions. PLOS Comput. Biol. 13:e1005714. 10.1371/journal.pcbi.1005714 28859079 PMC5597258

[B11] Coombes S. Timofeeva Y. (2003). Sparks and waves in a stochastic fire-diffuse-fire model of Ca^2+^ release. Phys. Rev. E Stat. Nonlin. Soft Matter Phys. 68:021915. 10.1103/PhysRevE.68.021915 14525014

[B12] Demir S. S. Clark J. W. Giles W. R. (1999). Parasympathetic modulation of sinoatrial node pacemaker activity in rabbit heart: a unifying model. Am. J. Physiol. Heart Circ. Physiol. 276, H2221–H2244. 10.1152/ajpheart.1999.276.6.H2221 10362707

[B13] Demir S. S. Clark J. W. Murphey C. R. Giles W. R. (1994). A mathematical model of a rabbit sinoatrial node cell. Am. J. Physiol. Cell Physiol. 266, C832–C852. 10.1152/ajpcell.1994.266.3.C832 8166247

[B14] Eijsbouts S. C. Majidi M. Zandvoort M. v Allessie M. A. (2003). Effects of acute atrial dilation on heterogeneity in conduction in the isolated rabbit heart. J. Cardiovasc. Electrophysiol. 14, 269–278. 10.1046/j.1540-8167.2003.02280.x 12716109

[B15] Frisk M. Koivumäki J. T. Norseng P. A. Maleckar M. M. Sejersted O. M. Louch W. E. (2014). Variable t-tubule organization and Ca^2+^ homeostasis across the atria. Am. J. Physiol. Heart Circ. Physiol. 307, H609–H620. 10.1152/ajpheart.00295.2014 24951751

[B16] Frommeyer G. Wolfes J. Ellermann C. Kochhäuser S. Dechering D. G. Eckardt L. (2019). Acute electrophysiologic effects of the polyphenols resveratrol and piceatannol in rabbit atria. Clin. Exp. Pharmacol. Physiol. 46, 94–98. 10.1111/1440-1681.13005 29956844

[B17] Greiser M. Kerfant B.-G. Williams G. S. Voigt N. Harks E. Dibb K. M.. (2014). Tachycardia-induced silencing of subcellular Ca^2+^ signaling in atrial myocytes. J. Clin. Invest. 124, 4759–4772. 10.1172/JCI70102 25329692 PMC4347234

[B18] Heijman J. Erfanian Abdoust P. Voigt N. Nattel S. Dobrev D. (2016). Computational models of atrial cellular electrophysiology and calcium handling, and their role in atrial fibrillation. J. Physiol. 594, 537–553. 10.1113/JP271404 26582329 PMC5341705

[B19] Hilgemann D. W. Matsuoka S. Nagel G. A. Collins A. (1992). Steady-state and dynamic properties of cardiac sodium-calcium exchange. Sodium-dependent inactivation. J. Gen. Physiol. 100, 905–932. 10.1085/jgp.100.6.905 1484285 PMC2229142

[B20] Hilgemann D. W. Noble D. (1987). Excitation-contraction coupling and extracellular calcium transients in rabbit atrium: reconstruction of basic cellular mechanisms. Proc. R. Soc. Lond. B Biol. Sci. 230, 163–205. 10.1098/rspb.1987.0015 2884668

[B21] Holmes M. Benson A. P. Aslanidi O. V. Colman M. A. (2018). “Investigating calcium-mediated arrhythmias via a computational model of a rabbit atrial myocyte,” in 2018 Computing in Cardiology Conference (CinC), Vol. 45 (Maastricht), 1–4. 10.22489/CinC.2018.188

[B22] Hou J.-W. Li W. Fei Y.-D. Chen Y.-H. Wang Q. Wang Y.-P.. (2018). I_*C*_*aL* and I_*t*_*o* mediate rate-dependent repolarization in rabbit atrial myocytes. J. Physiol. Biochem. 74, 57–67. 10.1007/s13105-017-0603-z 29243206

[B23] Hüser J. Lipsius S. L. Blatter L. A. (1996). Calcium gradients during excitation-contraction coupling in cat atrial myocytes. J. Physiol. 494, 641–651. 10.1113/jphysiol.1996.sp021521 8865063 PMC1160666

[B24] Johnstone R. H. Chang E. T. Bardenet R. de Boer T. P. Gavaghan D. J. Pathmanathan P.. (2016). Uncertainty and variability in models of the cardiac action potential: can we build trustworthy models? J. Mol. Cell. Cardiol. 96, 49–62. 10.1016/j.yjmcc.2015.11.018 26611884 PMC4915860

[B25] Kanaporis G. Blatter L. A. (2016). Calcium-activated chloride current determines action potential morphology during calcium alternans in atrial myocytes. J. Physiol. 594, 699–714. 10.1113/JP271887 26662365 PMC4930065

[B26] Kettlewell S. Saxena P. Dempster J. Colman M. A. Myles R. C. Smith G. L.. (2019). Dynamic clamping human and rabbit atrial calcium current: narrowing ical window abolishes early afterdepolarizations. J. Physiol. 597, 3619–3638. 10.1113/JP277827 31093979 PMC6767690

[B27] Kurata Y. Hisatome I. Imanishi S. Shibamoto T. (2002). Dynamical description of sinoatrial node pacemaking: improved mathematical model for primary pacemaker cell. Am. J. Physiol. Heart Circ. Physiol. 283, H2074–H2101. 10.1152/ajpheart.00900.2001 12384487

[B28] Ladd D. Tilunaité A. Roderick H. L. Soeller C. Crampin E. J. Rajagopal V. (2019). Assessing cardiomyocyte excitation-contraction coupling site detection from live cell imaging using a structurally-realistic computational model of calcium release. Front. Physiol. 10:1263. 10.3389/fphys.2019.01263 31632297 PMC6783691

[B29] Li H. Scherlag B. J. Kem D. C. Zillner C. Male S. Thirunavukkarasu S.. (2014). The propensity for inducing atrial fibrillation: a comparative study on old versus young rabbits. J. Aging Res. 2014:648918. 10.1155/2014/684918 24719763 PMC3955625

[B30] Lindblad D. Murphey C. Clark J. R. Giles W. (1996). A model of the action potential and underlying membrane currents in a rabbit atrial cell. Am. J. Physiol. 271, H1666–H1696. 10.1152/ajpheart.1996.271.4.H1666 8897964

[B31] Mackenzie L. Bootman M. D. Berridge M. J. Lipp P. (2001). Predetermined recruitment of calcium release sites underlies excitation-contraction coupling in rat atrial myocytes. J. Physiol. 530, 417–429. 10.1111/j.1469-7793.2001.0417k.x 11158273 PMC2278433

[B32] Mackenzie L. Roderick H. L. Berridge M. J. Conway S. J. Bootman M. D. (2004). The spatial pattern of atrial cardiomyocyte calcium signalling modulates contraction. J. Cell Sci. 117, 6327–6337. 10.1242/jcs.01559 15561771

[B33] Mahajan A. Shiferaw Y. Sato D. Baher A. Olcese R. Xie L.-H.. (2008). A rabbit ventricular action potential model replicating cardiac dynamics at rapid heart rates. Biophys. J. 94, 392–410. 10.1529/biophysj.106.98160 18160660 PMC2157228

[B34] Maltsev V. A. Lakatta E. G. (2009). Synergism of coupled subsarcolemmal Ca^2+^ clocks and sarcolemmal voltage clocks confers robust and flexible pacemaker function in a novel pacemaker cell model. Am. J. Physiol. Heart Circ. Physiol. 296, H594–H615. 10.1152/ajpheart.01118.2008 19136600 PMC2660239

[B35] Marchena M. Echebarria B. (2018). Computational model of calcium signaling in cardiac atrial cells at the submicron scale. Front. Physiol. 9:1760. 10.3389/fphys.2018.01760 30618786 PMC6295473

[B36] Marchena M. Echebarria B. (2020). Influence of the tubular network on the characteristics of calcium transients in cardiac myocytes. PLoS ONE 15:e0231056. 10.1371/journal.pone.0231056 32302318 PMC7164608

[B37] Morotti S. Grandi E. (2017). Logistic regression analysis of populations of electrophysiological models to assess proarrythmic risk. MethodsX 4, 25–34. 10.1016/j.mex.2016.12.002 28116246 PMC5225282

[B38] Muraki K. Imaizumi Y. Watanabe M. Habuchi Y. Giles W. R. (1995). Delayed rectifier K^+^ current in rabbit atrial myocytes. Am. J. Physiol. Heart Circ. Physiol. 269, H524–H532. 10.1152/ajpheart.1995.269.2.H524 7653616

[B39] Muszkiewicz A. Liu X. Bueno-Orovio A. Lawson B. A. J. Burrage K. Casadei B.. (2018). From ionic to cellular variability in human atrial myocytes: an integrative computational and experimental study. Am. J. Physiol. Heart Circ. Physiol. 314, H895–H916. 10.1152/ajpheart.00477.2017 29351467 PMC6008144

[B40] Nivala M. Song Z. Weiss J. N. Qu Z. (2015). T-tubule disruption promotes calcium alternans in failing ventricular myocytes: mechanistic insights from computational modeling. J. Mol. Cell Cardiol. 79, 32–41. 10.1016/j.yjmcc.2014.10.018 25450613 PMC4323351

[B41] Qi A. Yeung-Lai-Wah J. A. Xiao J. Kerr C. R. (1994). Regional differences in rabbit atrial repolarization: importance of transient outward current. Am. J. Physiol. Heart Circ. Physiol. 266, H643–H649. 10.1152/ajpheart.1994.266.2.H643 8141365

[B42] Rajagopal V. Bass G. Walker C. G. Crossman D. J. Petzer A. Hickey A.. (2015). Examination of the effects of heterogeneous organization of RYR clusters, myofibrils and mitochondria on Ca^2+^ release patterns in cardiomyocytes. PLoS Comput. Biol. 11:e1004417. 10.1371/journal.pcbi.1004417 26335304 PMC4559435

[B43] Ravelli F. Allessie M. (1997). Effects of atrial dilatation on refractory period and vulnerability to atrial fibrillation in the isolated langendorff-perfused rabbit heart. Circulation 96, 1686–1695. 10.1161/01.CIR.96.5.1686 9315565

[B44] Richards M. A. Clarke J. D. Saravanan P. Voigt N. Dobrev D. Eisner D. A.. (2011). Transverse tubules are a common feature in large mammalian atrial myocytes including human. Am. J. Physiol. Heart Circ. Physiol. 301, H1996–H2005. 10.1152/ajpheart.00284.2011 21841013 PMC3213978

[B45] Rouge L. Yoho J. Hayes K. Herling I. Gambert S. Ostrander G. K. (2006). 47 inefficacy of acetylcholine for induction of atrial fibrillation in rabbits. J. Invest. Med. 54, S264–S264. 10.2310/6650.2005.X0008.46

[B46] Sánchez C. Bueno-Orovio A. Wettwer E. Loose S. Simon J. Ravens U.. (2014). Inter-subject variability in human atrial action potential in sinus rhythm versus chronic atrial fibrillation. PLoS ONE 9:e0105897. 10.1371/journal.pone.0105897 25157495 PMC4144914

[B47] Sarkar A. X. Sobie E. A. (2010). Regression analysis for constraining free parameters in electrophysiological models of cardiac cells. PLoS Comput. Biol. 6:e1000914. 10.1371/journal.pcbi.1000914 20824123 PMC2932676

[B48] Shannon T. R. Wang F. Puglisi J. Weber C. Bers D. M. (2004). A mathematical treatment of integrated ca dynamics within the ventricular myocyte. Biophys. J. 87, 3351–3371. 10.1529/biophysj.104.047449 15347581 PMC1304803

[B49] Sher A. A. Noble P. J. Hinch R. Gavaghan D. J. Noble D. (2008). The role of the Na^+^/Ca^2+^ exchangers in Ca^2+^ dynamics in ventricular myocytes. Prog. Biophys. Mol. Biol. 96, 377–398. 10.1016/j.pbiomolbio.2007.07.018 17959231

[B50] Smyrnias I. Mair W. Harzheim D. Walker S. A. Roderick H. L. Bootman M. D. (2010). Comparison of the T-tubule system in adult rat ventricular and atrial myocytes, and its role in excitation–contraction coupling and inotropic stimulation. Cell Calcium 47, 210–223. 10.1016/j.ceca.2009.10.001 20106523

[B51] Song Z. Liu M. B. Qu Z. (2018). Transverse tubular network structures in the genesis of intracellular calcium alternans and triggered activity in cardiac cells. J. Mol. Cell. Cardiol. 114, 288–299. 10.1016/j.yjmcc.2017.12.003 29217432 PMC5801147

[B52] Sutanto H. van Sloun B. Schonleitner P. van Zandvoort M. A. M. J. Antoons G. Heijman J. (2018). The subcellular distribution of ryanodine receptors and L-Type Ca^2+^ channels modulates Ca^2+^-transient properties and spontaneous Ca^2+^-release events in atrial cardiomyocytes. Front Physiol 9, 1108. 10.3389/fphys.2018.01108 30166973 PMC6107030

[B53] Thul R. Coombes S. Roderick H. L. Bootman M. D. (2012). Subcellular calcium dynamics in a whole-cell model of an atrial myocyte. Proc. Natl. Acad. Sci. U.S.A. 109, 2150–2155. 10.1073/pnas.1115855109 22308396 PMC3277557

[B54] Tidball J. Cederdahl J. Bers D. (1991). Quantitative analysis of regional variability in the distribution of transverse tubules in rabbit myocardium. Cell Tissue Res. 264, 293–298. 10.1007/BF00313966 1715241

[B55] Trafford A. W. Clarke J. D. Richards M. A. Eisner D. A. Dibb K. M. (2013). Calcium signalling microdomains and the t-tubular system in atrial mycoytes: potential roles in cardiac disease and arrhythmias. Cardiovasc. Res. 98, 192–203. 10.1093/cvr/cvt018 23386275

[B56] Vagos M. van Herck I. G. M. Sundnes J. Arevalo H. J. Edwards A. G. Koivumäki J. T. (2018). Computational modeling of electrophysiology and pharmacotherapy of atrial fibrillation: Recent advances and future challenges. Front. Physiol. 9:1221. 10.3389/fphys.2018.01221 30233399 PMC6131668

[B57] Voigt N. Heijman J. Qiongling C.hiang, D. Y. Li N. Karck M. Wehrens X. H.. (2014). Cellular and molecular mechanisms of atrial arrhythmogenesis in patients with paroxysmal atrial fibrillation. Circulation 129, 145–156. 10.1161/CIRCULATIONAHA.113.006641 24249718 PMC4342412

[B58] Voigt N. Li N. Wang Q. Wang W. Trafford A. W. Abu-Taha I.. (2012). Enhanced sarcoplasmic reticulum Ca^2+^ leak and increased Na^+^-Ca^2+^ exchanger function underlie delayed afterdepolarizations in patients with chronic atrial fibrillation. Circulation 125, 2059–2070. 10.1161/CIRCULATIONAHA.111.067306 22456474 PMC4663993

[B59] Wang H. L. Zhou X. H. Li Z. Q. Fan P. Zhou Q. N. Li Y. D.. (2017). Prevention of atrial fibrillation by using sarcoplasmic reticulum calcium atpase pump overexpression in a rabbit model of rapid atrial pacing. Med. Sci. Monit. 23, 3952–3960. 10.12659/MSM.904824 28811460 PMC5569926

[B60] Wang Z. Fermini B. Feng J. Nattel S. (1995). Role of chloride currents in repolarizing rabbit atrial myocytes. Am. J. Physiol. Heart Circ. Physiol. 268, H1992–H2002. 10.1152/ajpheart.1995.268.5.H1992 7771549

[B61] Weber C. R. Ginsburg K. S. Philipson K. D. Shannon T. R. Bers D. M. (2001). Allosteric regulation of Na/Ca exchange current by cytosolic Ca in intact cardiac myocytes. J. Gen. Physiol. 117, 119–131. 10.1085/jgp.117.2.119 11158165 PMC2217247

[B62] Xie Y. Liao Z. Grandi E. Shiferaw Y. Bers D. M. (2015). Slow [Na]i changes and positive feedback between membrane potential and [Ca]i underlie intermittent early afterdepolarizations and arrhythmias. Circ. Arrhythm. Electrophysiol. 8, 1472–1480. 10.1161/CIRCEP.115.003085 26407967 PMC4681658

[B63] Yamashita T. Nakajima T. Hazama H. Hamada E. Murakawa Y. Sawada K.. (1995). Regional differences in transient outward current density and inhomogeneities of repolarization in rabbit right atrium. Circulation 92, 3061–3069. 10.1161/01.CIR.92.10.3061 7586277

[B64] Zaniboni M. (2011). 3D current-voltage-time surfaces unveil critical repolarization differences underlying similar cardiac action potentials: a model study. Math. Biosci. 233, 98–110. 10.1016/j.mbs.2011.06.008 21781977

