# Supplementary material for: A Novel Computational Model of the Rabbit Atrial Cardiomyocyte With Spatial Calcium Dynamics
Source: Front Physiol. 2020 Oct 9;11:556156. doi: 10.3389/fphys.2020.556156 (PMC7583320; doi:10.3389/fphys.2020.556156)
Supplement: Supplementary file 1 [file Data_Sheet_1.pdf]

Supplementary Material

1 NA$^+$-CA^{2+} EXCHANGER MODEL

$$I_{NCX} = I_{NCX}^{max} \cdot \frac{1}{1+\left(\frac{k_{dact}}{Ca_i}\right)^2} \cdot \frac{e^{\frac{\nu V_m F}{RT}} \cdot Ca_o \cdot Na_i^3 - e^{\frac{(\nu-1)V_m F}{RT}} \cdot Na_o^3 \cdot Ca_i}{\begin{array}{c} Km_{Ca_i} \cdot Na_o^3 \left(1+\left(\frac{Na_i}{Km_{Na_i}}\right)^3\right) \\ +Km_{Na_o}^3 \cdot Ca_i \cdot \left(1+\frac{Ca_i}{Km_{Ca_i}}\right) \\ +(Km_{Ca_o}+Ca_o)\cdot Na_i^3 + Na_o^3 \cdot Ca_i \end{array}} \cdot \frac{1}{1+k_{sat}\cdot e^{\frac{(\nu-1)V_m F}{RT}}}$$

$I_{\mathrm{NCX}}^{max} = 4.41\,\mathrm{pA/pF}$

$k_{dact} = 0.384 \times 10^{-3}\,\mathrm{mM}$

$Km_{Ca_i} = 3.59 \times 10^{-3}\,\mathrm{mM}$

$Km_{Ca_o} = 1.3\,\mathrm{mM}$

$Km_{Na_i} = 12.29\,\mathrm{mM}$

$Km_{Na_o} = 87.5\,\mathrm{mM}$

$nu = 0.35$

$k_{sat} = 0.27$

2 CA^{2+} AND NA$^+$ DIFFUSION MODEL

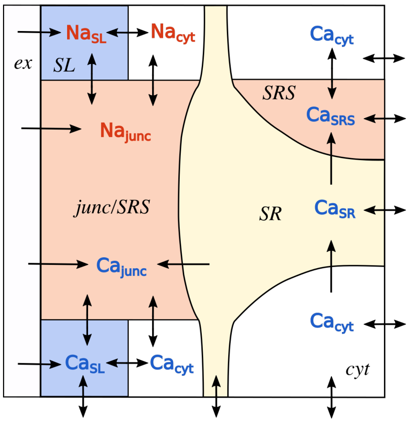

Figure S1. Schematic representation of Ca^{2+} and Na$^+$ concentrations and their across the different model compartments in a membrane domain.

3 NA$^+$ MASS BALANCE

In the case of the simplified

$$\frac{dNa_i}{dt} = \frac{J_{Na_{SLmyo}}}{v_{myo}} \cdot \Big((Na^1_{SL} - Na_i) + (Na^2_{SL} - Na_i) \Big) + \frac{(Na_i^{-1} - Na_i) + (Na_i^{-2} - Na_i)}{\tau_D}$$

where τ_D =0.6 /s is the diffusion constant. Na^1_{SL} and Na^2_{SL} are the Na$^+$ concentrations in the SL space of the membrane domains, and Na_i^{-1} and Na_i^{-2} are the Na$^+$ concentrations in the neighbouring left and right segments, respectively. $J_{Na_{slmoy}}$ is the net buffered Na$^+$ flux and equals 1.6386×10^{-12} L/ms.

Since in this study we simulate only one segment and both membrane domains are identical, the above equation simplifies to:

$$\frac{dNa_i}{dt} = \frac{J_{Na_{SLmyo}}}{v_{myo}} \cdot 2 \cdot \Big(Na_{SL} - Na_i \Big)$$

At the membrane domains, Na$^+$ is updated as:

$$\frac{dNa_{SL}}{dt} = I^{tot}_{Na_{SL}} \cdot \frac{C_m F}{v_{SL}} + J_{Na_{juncSL}} \cdot (Na_{junc} - Na_{SL}) + \frac{2 \cdot J_{Na_{SLmyo}}}{v_{SL}} \cdot (Na_i - Na_{SL}) - Buff_{Na_{SL}}$$

$$\frac{dNa_{junc}}{dt} = I^{tot}_{Na_{junc}} \cdot \frac{C_m F}{v_{junc}} + \frac{J_{Na_{juncSL}}}{V_{junc}} \cdot (Na_{SL} - Na_{junc}) - Buff_{Na_{junc}}$$

where $J_{Na_{juncSL}}$ =1.8313×10^{-14} l/ms is the Na$^+$ flux between the SL and junc spaces. $Buff_{Na_{SL}}$ and $Buff_{Na_{junc}}$ are the buffering variables of Na$^+$ in the SL and junc spaces, respectively, and are given by:

$$\frac{dBuff_{Na_{SL}}}{dt} = k^{Na}_{on} \cdot Na_{SL} \cdot (B^{max}_{Na_{SL}} - Buff_{Na_{SL}}) - k^{Na}_{off} \cdot Buff_{Na_{SL}}$$

$$\frac{dBuff_{Na_{junc}}}{dt} = k^{Na}_{on} \cdot Na_{junc} \cdot (B^{max}_{Na_{junc}} - Buff_{Na_{junc}}) - k^{Na}_{off} \cdot Buff_{Na_{junc}}$$

where $k^{Na}_{on} = 0.0001$ and $k^{Na}_{off} = 0.001$ are the rate constants of Na$^+$buffer complex formation and dissociation, respectively. $B^{max}_{Na_{SL}}$ and $B^{max}_{Na_{junc}}$ are the maximum buffer capacities in the SL and junc spaces and are equal to 7.561 mM and 1.65 mM, respectively.

4 SERCA2A MODEL

$$J_{Serca} = V_{max} \cdot \frac{\left(\frac{Ca_i}{Kmf}\right)^{nh} - \left(\frac{Ca_{SR}}{Kmr}\right)^{nh}}{1 + \left(\frac{Ca_i}{Kmf}\right)^{nh} + \left(\frac{Ca_{SR}}{Kmr}\right)^{nh}}$$

$V_{max} = 5.3 \times 10^{-3}\,\mathrm{mM/s}$

$Kmf = 0.000\,625\,\mathrm{mM}$

$Kmr = 1.0\,\mathrm{mM}$

$nh = 1.787$

5 CA^{2+} BUFFERS

Buffers are modelled using a two-state model:

$$\frac{dC_B}{dt} = k_{on}C(B_{max} - C_B) - k_{off}C_B$$

where B_{max} is the maximum buffer capacity, C and Ca_B represent the concentration of free and bound ligand, and k_{on} and k_{off} are the rate coefficients of ion-buffer complex formation and dissociation, respectively.

Table S1. Buffer parameters.

**Buffer**	**Compartment**	**Bmax** (µM)	**kon** (/s)	**koff** (/µMs)
Troponin C High (Ca^{2+})	cytosol	140E-3	2.37	3.2E-5
Troponin C High (Mg^{2-})	cytosol	140E-3	3E-3	3.33E-3
Troponin C Low	cytosol	70E-3	163.5	98E-3
Calmodulin	cytosol	2.4E-3	34	238E-3
Myosin (Ca)	cytosol	140E-3	13.8	4.6E-4
Myosin (Mg)	cytosol	140E-3	0.0157	5.7E-5
SR	cytosol	17.1E-3	100	6E-2
SLlow	SL	37.4E-3$\cdot\frac{v_{myo}}{v_{SL}}$	100	1.3
SLlow	junc	4.6E-4$\cdot\frac{v_{myo}}{v_{junc}}$	100	1.3
SLhigh	SL	13.4E-3$\cdot\frac{v_{myo}}{v_{SL}}$	100	3E-2
SLhigh	junc	1.65E-4$\cdot\frac{v_{myo}}{v_{junc}}$	100	3E-2
Calsequestrin	SR	140E-3$\cdot\frac{v_{myo}}{v_{SR}}$	100	65
Na^{+}	SL	7.561	1.0E-4	1.0E-3
Na^{+}	junc	1.65	1.0E-4	1.0E-3

6 INITIAL CONDITIONS

Table S2. Initial ionic concentrations in the different compartments of the model.

Ionic traces	Initial concentration	Description
Ca_{cyt}	0.21 µM	Ca^{2+}concentration in the cytosol.
Ca_{junc}	0.073 µM	Ca^{2+} concentration in the junctional domain of the SS space.
Ca_{sl}	0.23 µM	Ca^{2+} concentration in the sarcolemmal domain of the SS space.
Ca_{SR}	0.25 µM	Luminal Ca^{2+} concentration in the sarcoplasmic reticulum.
Ca_{SRS}	0.073 µM	Ca^{2+} concentration in the sub SR space.
Na_{cyt}	11.3 mM	Na^{+} concentration in the cytosol (2Hz).
Na_{junc}	11.3 mM	Na^{+} concentration in the junctional domain of the SS space.
Na_{SL}	11.3 mM	Na^{+} concentration in the sarcolemmal domain of the SS space.
K_{cyt}	120 mM	K^{+} concentration in the cytosol.
Cl_{cyt}	30 mM	Cl^{-} concentration in the cytosol.
Ca_{o}	1.8 mM	Extracellular Ca^{2+} concentration.
Na_{o}	140 mM	Extracellular Na^{+} concentration.
K_{o}	5.4 mM	Extracellular K^{+} concentration.
Cl_{o}	132 mM	Extracellular Cl^{-} concentration.

7 SELECTED POPULATION

Table S3. Parameter values of the 16 models of the calibrated population.

G_{CaL}	G_{CaT}	I_{NaK}^{max}	I_{NaCa}^{max}	G_{Na}	G_{to}	G_{Kr}	G_{Ks}	G_{K1}	I_{CaP}^{max}	G_{Cab}	G_{Nab}	G_{Clb}
0.228	0.415	2.449 E-3	1.436	0.088 E-3	0.712	0.120	0.174	0.623	0.589	0.970 E-3	0.398 E-3	6.392 E-3
0.146	0.474	1.801 E-3	1.162	0.068 E-3	0.606	0.056	0.119	0.333	0.246	0.506 E-3	0.623 E-3	7.647 E-3
0.192	0.069	2.425 E-3	1.073	0.068 E-3	0.443	0.268	0.058	0.277	0.401	1.348 E-3	0.885 E-3	5.078 E-3
0.202	0.437	1.651 E-3	3.314	0.060 E-3	0.492	0.176	0.178	0.673	0.403	1.024 E-3	0.612 E-3	8.786 E-3
0.295	0.124	2.573 E-3	1.165	0.071 E-3	0.790	0.248	0.199	0.263	0.244	0.348 E-3	0.631 E-3	5.742 E-3
0.099	0.147	1.320 E-3	0.762	0.063 E-3	0.155	0.135	0.182	0.344	0.466	1.022 E-3	0.772 E-3	4.047 E-3
0.256	0.156	1.892 E-3	3.786	0.050 E-3	0.354	0.193	0.155	0.777	0.067	1.033 E-3	0.143 E-3	6.964 E-3
0.189	0.406	1.599 E-3	1.313	0.064 E-3	0.435	0.181	0.084	0.697	0.582	0.482 E-3	0.944 E-3	5.345 E-3
0.222	0.325	2.366 E-3	2.012	0.104 E-3	0.449	0.209	0.122	0.625	0.395	1.594 E-3	0.221 E-3	5.321 E-3
0.189	0.431	2.100 E-3	1.532	0.042 E-3	0.514	0.205	0.113	0.432	0.220	1.360 E-3	0.176 E-3	7.643 E-3
0.231	0.038	1.602 E-3	3.161	0.073 E-3	0.604	0.142	0.174	0.299	0.128	0.524 E-3	0.405 E-3	7.133 E-3
0.322	0.388	2.407 E-3	3.200	0.109 E-3	0.558	0.262	0.121	0.414	0.315	0.949 E-3	1.570 E-3	8.421 E-3
0.180	0.458	1.934 E-3	1.081	0.062 E-3	0.162	0.144	0.062	0.314	0.455	0.312 E-3	0.851 E-3	7.995 E-3
0.293	0.204	2.671 E-3	1.528	0.104 E-3	0.729	0.262	0.181	0.396	0.505	0.173 E-3	1.228 E-3	7.827 E-3
0.201	0.305	1.302 E-3	1.882	0.105 E-3	0.699	0.161	0.133	0.531	0.263	0.471 E-3	0.112 E-3	8.358 E-3
0.155	0.329	1.775 E-3	3.535	0.094 E-3	0.574	0.146	0.129	0.380	0.339	1.410 E-3	1.373 E-3	3.252 E-3

8 I-V CURVES

Table S4. Maximum and minimum peak current values obtained from simulated I-V curves in Fig. S2, and comparison with experimental values reported in Aslanidi et al. (2009)(1) and Muraki et al. (1995)(2). Values are in pA/pF.

	**I-V curves**			**2 Hz Pacing**		
	**Simulated**		**Experimental**	**Our model**		**Aslanidi model**
	**Min. peak**	**Max. peak**		**Min. peak**	**Max. peak**	
I_{CaL}	−13.9	−4.3	−8 (PM), −6 (CT)	−6.4	−2.1	−3.6
I_{Na}	−737	−291	−70	−393	−192	−120
I_{to}	3.4	16.6	13 (PM), 8 (CT)	0.87	4.0	1.3
I_{K1}	0.88	2.6	5 (PM), 4 (CT)	0.88	2.6	0.66
I_{Kr}	0.45	2.1	0.67*	0.41	2.4	0.43

* Assuming a cell capacitance of $50\,pF$

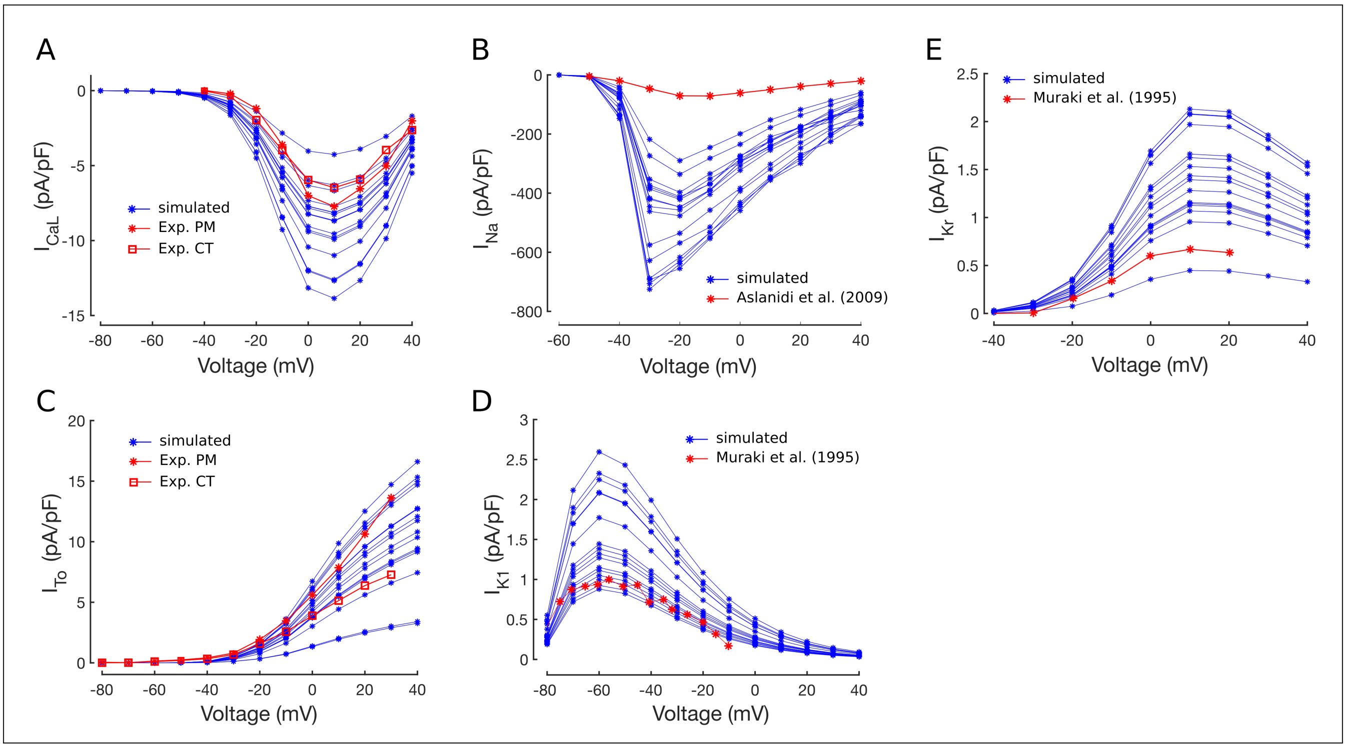

Figure S2. Simulated I-V curves (blue) of I_{CaL}, I_{Na}, I_{to}, and I_{K1} for the 16 models in the control population. The voltage-clamp protocol applied consisted of trains of $200\,\text{ms}$ square depolarizing pulses at a cycle length of $2\,\text{s}$ from a holding potential of $-80\,\text{mV}$ to voltages between $-80\,\text{mV}$ and $40\,\text{mV}$, in steps of $10\,\text{mV}$. Experimental I-V curves (red) are shown for comparison. In panels A and C, curves for both pectinate muscle (PM, stars) and crista terminalis (CT, squares) rabbit atrial CMs are showm (data from Aslanidi et al. (2009)). The experimental curve in panel D is normalized data, shown here for qualitative comparison purposes. The experimental curve in E assumed a cell capacitance of $50\,\text{pF}$.

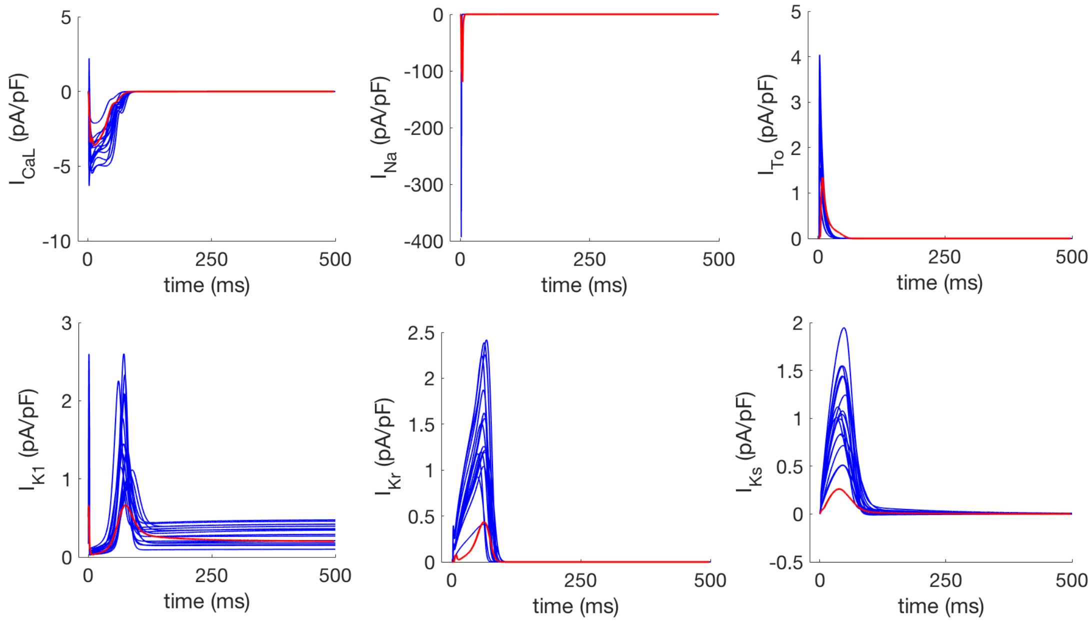

Figure S3. Comparison between I_{CaL}, I_{Na}, I_{to}, I_{K1}, I_{Kr}, and I_{Ks} obtained with our control population and with the Aslanidi model(1) (CellML version) at $2\,\text{Hz}$ pacing.

REFERENCES

[1]Aslanidi et al. *Mechanisms of transition from normal to reentrant electrical activity in a model of rabbit atrial tissue: Interaction of tissue heterogeneity and anisotropy*, Biophysical Journal 96(3), 798-817, 2009.

[2]Muraki et al. *Delayed rectifier K^+ current in rabbit atrial myocytes*, Am J of Phys-Heart and Circ Phys 269(2), H524-H532, 1995.
